# WNK1 regulates uterine homeostasis and its ability to support pregnancy

**DOI:** 10.1172/jci.insight.141832

**Published:** 2020-11-19

**Authors:** Ru-pin Alicia Chi, Tianyuan Wang, Chou-Long Huang, San-pin Wu, Steven L. Young, John P. Lydon, Francesco J. DeMayo

**Affiliations:** 1 Reproductive and Developmental Biology Laboratory and; 2 Integrative Bioinformatics Support Group, National Institute of Environmental Health Sciences, NIH, Durham, North Carolina, USA.; 3 Department of Internal Medicine, University of Iowa Carver College of Medicine, Iowa, Iowa, USA.; 4 Department of Obstetrics and Gynecology, University of North Carolina at Chapel Hill, Chapel Hill, North Carolina, USA.; 5 Department of Molecular and Cellular Biology, Baylor College of Medicine, Houston, Texas, USA.

**Keywords:** Reproductive Biology, Fertility, Obstetrics/gynecology, Phosphoprotein phosphatases

## Abstract

WNK1 (with no lysine [K] kinase 1) is an atypical kinase protein ubiquitously expressed in humans and mice. A mutation in its encoding gene causes hypertension in humans, which is associated with abnormal ion homeostasis. WNK1 is critical for in vitro decidualization in human endometrial stromal cells, thereby demonstrating its importance in female reproduction. Using a mouse model, WNK1 was ablated in the female reproductive tract to define its in vivo role in uterine biology. Loss of WNK1 altered uterine morphology, causing endometrial epithelial hyperplasia, adenomyotic features, and a delay in embryo implantation, ultimately resulting in compromised fertility. Combining transcriptomic, proteomic, and interactomic analyses revealed a potentially novel regulatory pathway whereby WNK1 represses AKT phosphorylation through protein phosphatase 2A (PP2A) in endometrial cells from both humans and mice. We show that WNK1 interacted with PPP2R1A, the alpha isoform of the PP2A scaffold subunit. This maintained the levels of PP2A subunits and stabilized its activity, which then dephosphorylated AKT. Therefore, loss of WNK1 reduced PP2A activity, causing AKT hypersignaling. Using FOXO1 as a readout of AKT activity, we demonstrate that there was escalated FOXO1 phosphorylation and nuclear exclusion, leading to a disruption in the expression of genes that are crucial for embryo implantation.

## Abstract 2

The atypical kinase WNK1 is critical for maintaining uterine homeostasis and mediating proper implantation through the PP2A/AKT signaling pathway.

## Introduction

Aberrant embryo implantation results in a ripple effect leading to pregnancy complications and miscarriages (1). Embryo implantation occurs during the “window of receptivity” and requires a fully prepared and responsive uterus. WNK1 (with no lysine [K] kinase 1) has been identified as a potential regulator of uterine function acting downstream of EGFR that regulates endometrial stroma cell proliferation, migration, and differentiation in vitro (2, 3). These findings indicate a previously unrecognized function of WNK1 in the female reproductive tract and led us to hypothesize that WNK1 is a mediator of uterine function.

WNK1 belongs to a family of serine/threonine protein kinases (4, 5), with its name derived from the unusual placement of the catalytic lysine in subdomain I (6). To date, WNK1’s function has been most extensively explored in the kidney and the nervous system because of the link between its mutation and familial hypertension and autonomic neuropathy (7–9). In the renal system, WNK1 controls ion homeostasis through diverse mechanisms, including activation of the SGK1/epithelial sodium channel pathway (10), regulating the potassium channel Kir1.1 cell surface localization (11), as well as controlling the activity of Na-K-Cl cotransporter through phosphorylating oxidative stress responsive kinase 1 (OXSR1/OSR1) and SPAK (12, 13). Interestingly, WNK1’s regulatory function on OSR1 is critical for cardiovascular development, thereby contributing to embryonic lethality when ablated from the endothelium (14, 15). These findings suggest that although WNK1 exhibits organ-specific physiological functions, the underlying cellular components regulated by WNK1 may share similarity between the different tissues.

Despite its ubiquitous expression, WNK1’s role in organs other than those described above remain unexplored. Given the role of WNK1 in regulating uterine stromal cell biology in vitro, we hypothesized that WNK1 is essential in regulating uterine functions. To test this, we genetically ablated *Wnk1* in the reproductive tract of female mice and demonstrated that WNK1 is critical in maintaining uterine morphology, regulating epithelial proliferation, and permitting appropriate embryo implantation. Transcriptomic and proteomic analyses identified deregulation of the AKT signaling pathway underlying the observed phenotypes. Using cultured human endometrial cells, we conducted a series of functional analyses to tease out the mechanisms of WNK1’s function in uterine biology.

## Results

### WNK1 is expressed in the uterus during the window of implantation in both humans and mice.

**Figure 1:**
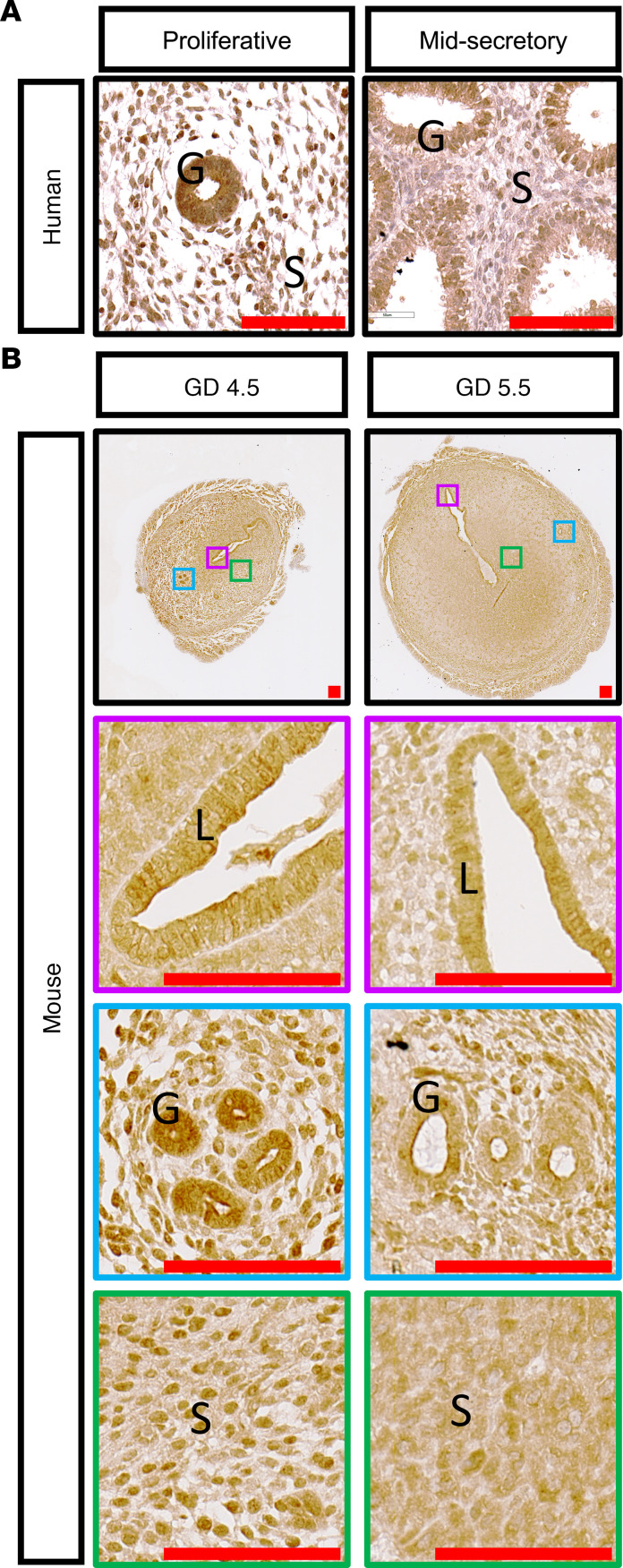
WNK1 was expressed in all compartments of the uterus during the window of implantation in both humans and mice. (**A** and **B**) IHC staining of WNK1 in proliferative and midsecretory phased endometrial tissues from fertile women (**A**), and during receptive GD 4.5 and after implantation/decidualizing phase GD 5.5 in the uterus of WT mice, with the colored squares indicating positions of enlarged areas (**B**). Scale bars: 100 μm. G, glandular epithelium; S, stroma; L, luminal epithelium.

WNK1 expression was examined by IHC in human endometrium during the proliferative and midsecretory phases as well as in the peri-implantation uterus of mice. In humans, WNK1 is expressed in both the epithelium and stroma during the proliferative and midsecretory phases (Figure 1A). Similarly in mice, WNK1 is expressed during and after implantation on gestation days (GDs) 4.5 and 5.5 (Figure 1B). These findings support the in vivo involvement of WNK1 in regulating functions of the female reproductive tract.

### WNK1 ablation altered uterine morphology and microenvironment.

To examine WNK1’s function in the female reproductive tract, we crossed conditional *Wnk1* allele mice (*Wnk1^fl/fl^*) to *PGR^Cre^* mice (14, 16) and showed that in the *PGR^Cre/+^* *Wnk1^fl/fl^* offspring (*Wnk1^d/d^*), *Cre*-mediated excision led to the removal of exon 2 (Supplemental Figure 1, A and B; supplemental material available online with this article; https://doi.org/10.1172/jci.insight.141832DS1). The whole-uterus WNK1 level was confirmed to be reduced in the *Wnk1^d/d^* uteri (Supplemental Figure 1C), by 7.1-fold according to densitometrical quantification of the signal intensity. The WNK1 expression detected in the *Wnk1^d/d^* mice was due to immune and endothelial cells, which do not express PGR.

**Figure 2:**
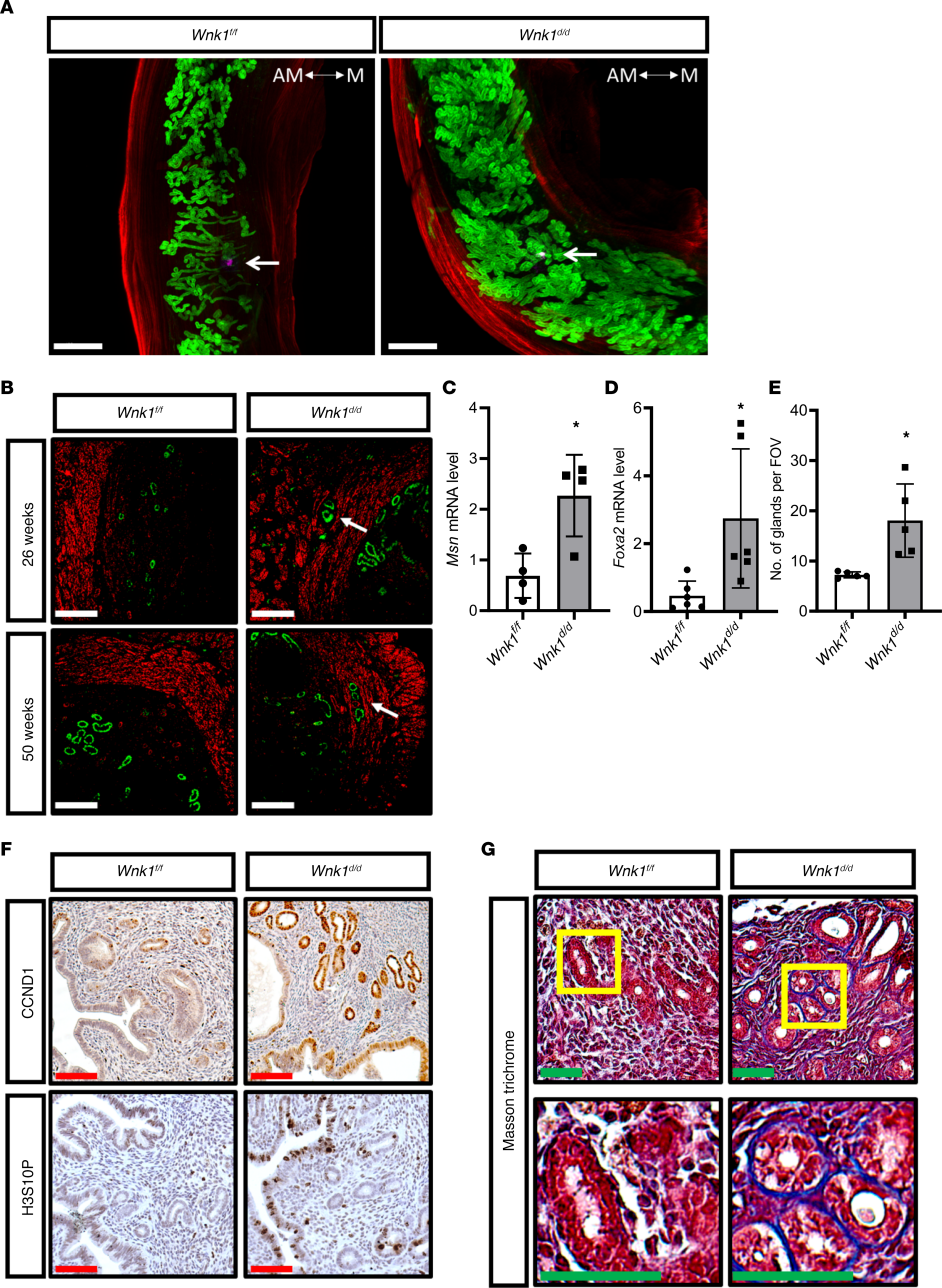
WNK1 ablation altered uterine morphology and microenvironment. (**A**) 3D reconstruction of *Wnk1^fl/fl^* (*Wnk1^f/f^*) and *Wnk1^d/d^* uteri on GD 4.5 using tissue clearing and confocal microscopy. The glands, myometrium and embryo were marked by FOXA2 (green), ACTA2 (red), and OCT4 (purple), respectively. Images were captured by tile-scanning and *Z*-stacking and reassembled in silico using Imaris software. White arrow indicates position of the embryo. Scale bars: 500 μm. The antimesometrial (AM) and mesometrial (M) sides of the tissue are indicated. FOXA2, forkhead box A2; ACTA2, actin alpha 2, smooth muscle; OCT4, POU class 5 homeobox 1 (POU5F1/OCT4). (**B**) Immunofluorescence of uterine cross section showing glands (FOXA2, green) and myometrium (ACTA2, red) from *Wnk1^fl/fl^* and *Wnk1^d/d^* uteri. White arrows indicate gland extension into myometrium. Scale bars: 50 μm. (**C**) Adenomyosis biomarker *Msn* mRNA expression as determined by quantitative real-time PCR (qRT-PCR) (*n* = 4). (**D**) Quantification of *Foxa2* mRNA expression as determined by qRT-PCR (*n* = 6). (**E**) Number of glands per cross section for *Wnk1^fl/fl^* and *Wnk1^d/d^* mice (*n* = 6). (**F**) Expression of mitotic markers CCND1 and H3S10P in the uteri of 26-week-old *Wnk1^fl/fl^* and *Wnk1^d/d^* mice; scale bars: 100 μm. (**G**) Masson’s trichrome staining of uterine cross sections from 26- and 50-week-old *Wnk1^fl/fl^* and *Wnk1^d/d^* mice; scale bars: 100 μm. Yellow boxes indicate region shown at higher magnification in lower panels. All quantitative results shown are mean ± SD, *P < 0.05. All *t* tests were 2-tailed, Student’s *t* test (**C** and **E**), and Mann-Whitney *U* test (**D**).

A tissue clearing technique was employed to visualize uterine morphology in 3D during the window of receptivity (Figure 2A) (17). WNK1 deficiency caused an increased number and altered structure of the endometrial glands (Figure 2A). Among the abnormalities observed in the *Wnk1^d/d^* uteri was the failure of gland reorientation surrounding the embryo (18). This was seen in the *Wnk1^fl/fl^* uteri where glands near the embryo exhibited an elongated structure while glands away from the embryo remained tortuous and intertwined. In the *Wnk1^d/d^* uteri, the glands appeared equally tortuous irrespective of distance from embryo (Figure 2A). Examination of uterine cross sections from older mice (26 and 50 weeks) further demonstrated invasion of glands into the myometrium, suggesting that WNK1 ablation caused adenomyosis-like features (Figure 2B). This was supported by the elevated expression of *Moesin* (*Msn*) in the *Wnk1^d/d^* uteri, a biomarker for adenomyosis in humans (Figure 2C) (19). Quantification of gland number and *Foxa2* gene expression showed significant elevation in the *Wnk1^d/d^* uteri (Figure 2, D and E), confirming the substantial increase in glandular tissues. To examine whether the increase in glands was a result of increased proliferation in the uterus, we examined 2 mitotic markers, cyclin D1 (CCND1) and phosphorylated histone H3 (H3S10p). Elevated levels of both proteins in the glandular epithelium of the *Wnk1^d/d^* uteri were observed (Figure 2F). In addition, higher expression of both proteins was also observed in the luminal epithelium of *Wnk1^d/d^* uteri, demonstrating that WNK1 ablation–induced epithelial hyperplasia was not restricted to the glands but also was apparent in the luminal epithelium. Moreover, we observed increased extracellular matrix deposition, especially surrounding the glands in the *Wnk1^d/d^* uteri, as shown by Masson’s trichrome staining (Figure 2G). These results suggest that the adenomyotic phenotype could be associated with increased epithelial proliferation as well as excessive extracellular matrix deposition (20, 21).

### Uterine loss of WNK1 compromised fertility and impaired implantation.

**Figure 3:**
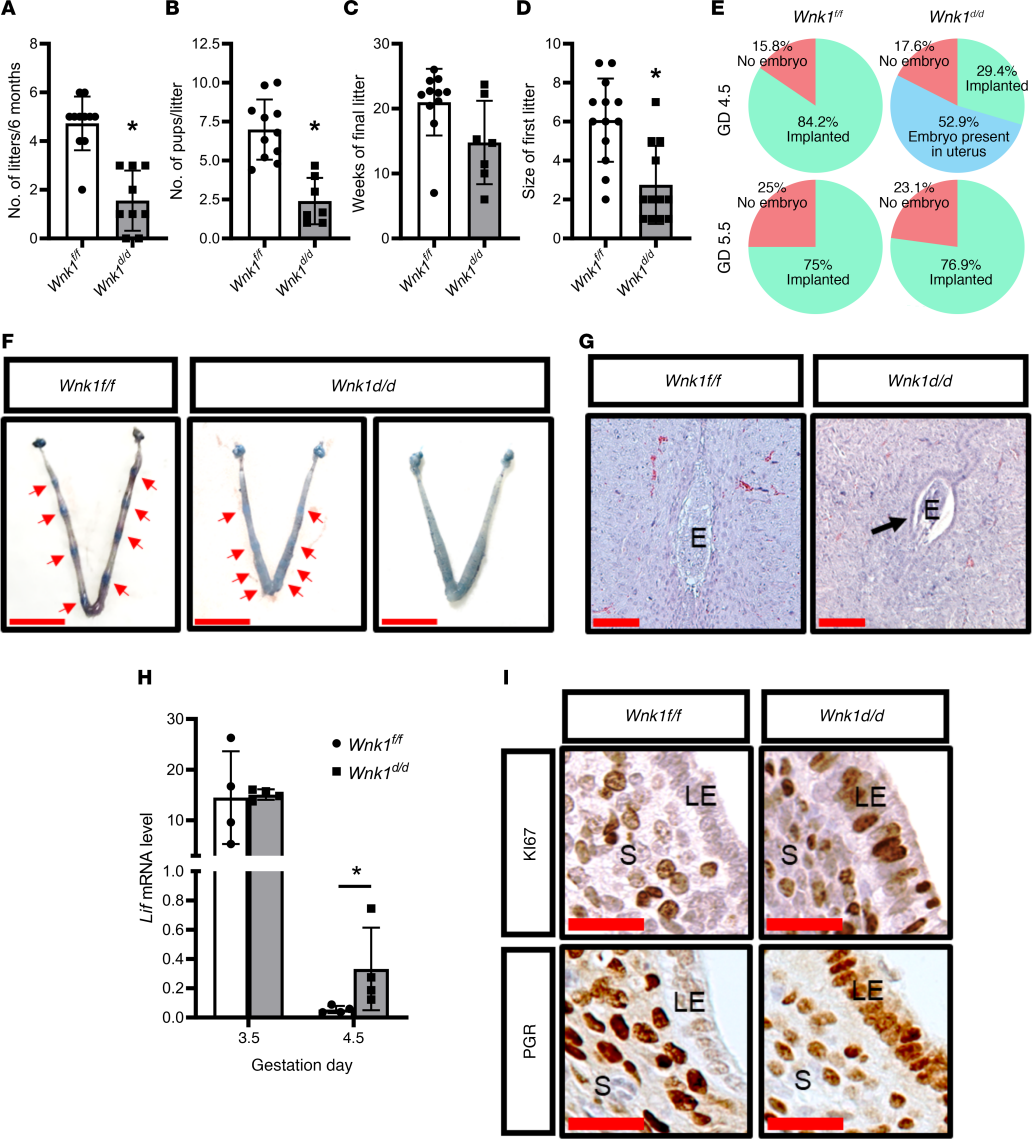
Uterine WNK1 ablation compromised fertility and impaired implantation in mice. (**A**–**D**) Results from a 6-month breeding trial, showing (**A**) average number of litters produced per mouse, (**B**) average number of pups per litter, (**C**) time of last delivery from the onset of the breeding trial, and (**D**) average size of the first litters. *n* = 11 for *Wnk1^fl/fl^* and *n* = 9 for *Wnk1^d/d^* mice (**A**), *n* = 11 for *Wnk1^fl/fl^* and *n* = 7 for *Wnk1^d/d^* (**B** and **C**), and *n* = 13 for *Wnk1^fl/fl^* and *n* = 12 for *Wnk1^d/d^* (**D**). Results shown are mean ± SD, *P < 0.05. (**E**) Percentage of mated *Wnk1^fl/fl^* and *Wnk1^d/d^* mice with implantation (green), without implantation (pink), and without implantation but presenting embryos in the uterus (blue) on GD 4.5 and GD 5.5. *n* = 19 and 12 for *Wnk1^fl/fl^* mice on GD 4.5 and GD 5.5, respectively; and *n* = 17 and 13 for *Wnk1^d/d^* mice on GD 4.5 and GD 5.5, respectively. (**F**) Gross uterine morphology of *Wnk1^fl/fl^* and *Wnk1^d/d^* mice on GD 4.5, with the implantation sites marked by Evans blue dye; scale bars: 1 cm. Red arrows indicate position of implantation sites. (**G**) Hematoxylin and eosin staining of uterine cross sections at implantation site on GD 5.5 in *Wnk1^fl/fl^* and *Wnk1^d/d^* mice; arrow indicates presence of uterine epithelium. Scale bars: 100 μm. E, embryo. (**H**) Implantation marker *Lif* mRNA expression in the uteri as determined by qRT-PCR on GD 3.5 and PPD 4.5 for *Wnk1^fl/fl^* and *Wnk1^d/d^* mice. Results shown are mean ± SD, *P < 0.05, *n* = 4. (**I**) Expression of proliferative marker KI67 and implantation marker PGR on GD 4.5 in the stroma and epithelium of *Wnk1^fl/fl^* and *Wnk1^d/d^* mice. Scale bars: 25 μm. All *t* tests were 2-tailed, Student’s *t* test (**B** and **H**), Mann-Whitney *U* test (**A**, **C**, and **D**), and Fisher’s exact test (**E**). LE, luminal epithelium; S, stroma.

A breeding trial was conducted to determine the impact of WNK1 ablation on female fertility. Here, both the *Wnk1^fl/fl^* and *Wnk1^d/d^* female mice were mated one-on-one to WT males for 6 months, during which the mice were closely monitored for pregnancy and delivery (Supplemental Table 1). Of the 13 control mice, 11 were able to complete the breeding trial (85%), with 2 found dead midtrial. Necropsy showed neither pregnancy nor abnormality associated with the reproductive tract in these 2 mice, indicating that the cause of death was not related to abnormal uterine function. The 11 mice produced 52 litters totaling 355 pups, which was equivalent to 5 litters and 6.8 pups per litter during the 6 months (Figure 3A). In contrast, only 9 of the 14 *Wnk1^d/d^* mice initiated in the trial were able to complete the trial (64%), owing to 5 females succumbing to complications during pregnancy or delivery. Of those, 2 were found dead near term each carrying 2 pups, and 2 were in dystocia and had to undergo euthanization. One was found ill and necropsy showed uteroabdominal fistula. Of the 9 females that completed the trial, 14 litters and 37 pups were produced, which averaged out to 1.6 litters and 2.6 pups per litter (Figure 3A). Additionally, of the 9 *Wnk1^d/d^* mice that survived to the end of the breeding trial, 2 never delivered, 3 delivered only 1 litter, and no mouse delivered beyond 3 litters (Figure 3A and Supplemental Table 1). The average litter size was also significantly smaller, averaging 2.4 pups per litter in these mice, compared with 7.0 in the control *Wnk1^fl/fl^* mice (Figure 3B). While the *Wnk1^fl/fl^* mice bred consistently, producing the last litters in or after the 20th week of the trial with only one exception, 5 of the 7 *Wnk1^d/d^* mice that reproduced stopped breeding before the 15th week (Figure 3C), indicating that there was premature loss of fertility. This loss of fertility was, however, not due to a progressive decrease in reproductive capacity, as demonstrated by the significantly smaller size of the first litters produced by the *Wnk1^d/d^* mice compared with their control siblings (Figure 3D). Taken together, these results illustrated compromised ability to support pregnancy and premature sterility with uterine loss of WNK1.

We next examined whether the subfertile phenotype was associated with an implantation defect in the *Wnk1^d/d^* mice. Dams were euthanized on GD 4.5, and embryo implantation was visualized by Evans blue dye staining. As expected, 84.2% of the control mice successfully permitted embryo implantation on GD 4.5 while the remaining 15.8% had no embryos present in the uterus (Figure 3E, top panel, and Figure 3F). Significantly fewer of the mated *Wnk1^d/d^* mice were able to form implantation sites on GD 4.5 (29.4%; *P* = 0.0019); however, 52.9% of the mice harbored fertilized embryos inside the uterus (Figure 3E, top panel, and Figure 3F). Examination of the uterus on GD 5.5 showed 76.9% of the *Wnk1^d/d^* mice with implantation sites, which was, at this time point, comparable to their *Wnk1^fl/fl^* control littermates (Figure 3E, bottom panel). Histological examination showed that the control *Wnk1^fl/fl^* mice had already degraded the epithelium, enabling the embryo to invade the underlying stroma (Figure 3G, left), while *Wnk1^d/d^* uteri had intact uterine epithelium at this time point with the embryo trapped inside the luminal space (Figure 3G, right). These findings demonstrate that implantation is severely delayed in the *Wnk1^d/d^* mice.

The *PGR^Cre^* mouse model also causes recombination in the ovaries and pituitary; therefore, we next evaluated the contribution of these tissues to the WNK1 ablation phenotype. Ovarian function was evaluated by assaying ovulation and ovarian steroid hormone levels. The mice were subjected to superovulatory regimen of gonadotropins, followed by mating to WT male mice, and euthanized on GD 1.5 to simultaneously monitor ovulation and fertilization of the oocytes. We found a subtle albeit nonsignificant decrease in the number of 2-cell embryos produced by the *Wnk1^d/d^* dams, indicating that ovulation and fertilization were not affected (Supplemental Figure 2A). Additionally, serum estradiol (E_2_) and progesterone (P_4_) levels were similar between the *Wnk1^fl/fl^* and *Wnk1^d/d^* mice on GD 4.5 (Supplemental Figure 2, B and C), demonstrating that the pituitary and ovaries were functional and able to produce and maintain hormone levels. Therefore, the main contributing factor for the delayed implantation was not a malfunction of the pituitary/ovarian axis.

Prerequisites for uterine receptivity are the production of leukemia inhibitory factor (LIF) from the uterine glands and the cessation of epithelial proliferation before implantation on GD 3.5 (22, 23), as well as suppression of epithelial PGR expression during implantation on GD 4.5 (24). Hence, we examined the uterus to see whether the delayed implantation was associated with impairment of those parameters. *Lif* expression on GD 3.5 was similarly induced in the *Wnk1^fl/fl^* and *Wnk1^d/d^* mice, suggesting that aberrant *Lif* expression was not the cause of disrupted implantation (Figure 3H). In the control mice, there was little to no expression of KI67 and PGR in the luminal epithelium on GD 4.5, as expected (Figure 3I). The *Wnk1^d/d^* mice, however, maintained the expression of both proteins during the window of implantation (Figure 3I), demonstrating that crucial implantation-associated molecular events were deregulated in the *Wnk1^d/d^* mice.

### Abnormal embryo development and increased resorption in Wnk1^d/d^ mice.

**Figure 4:**
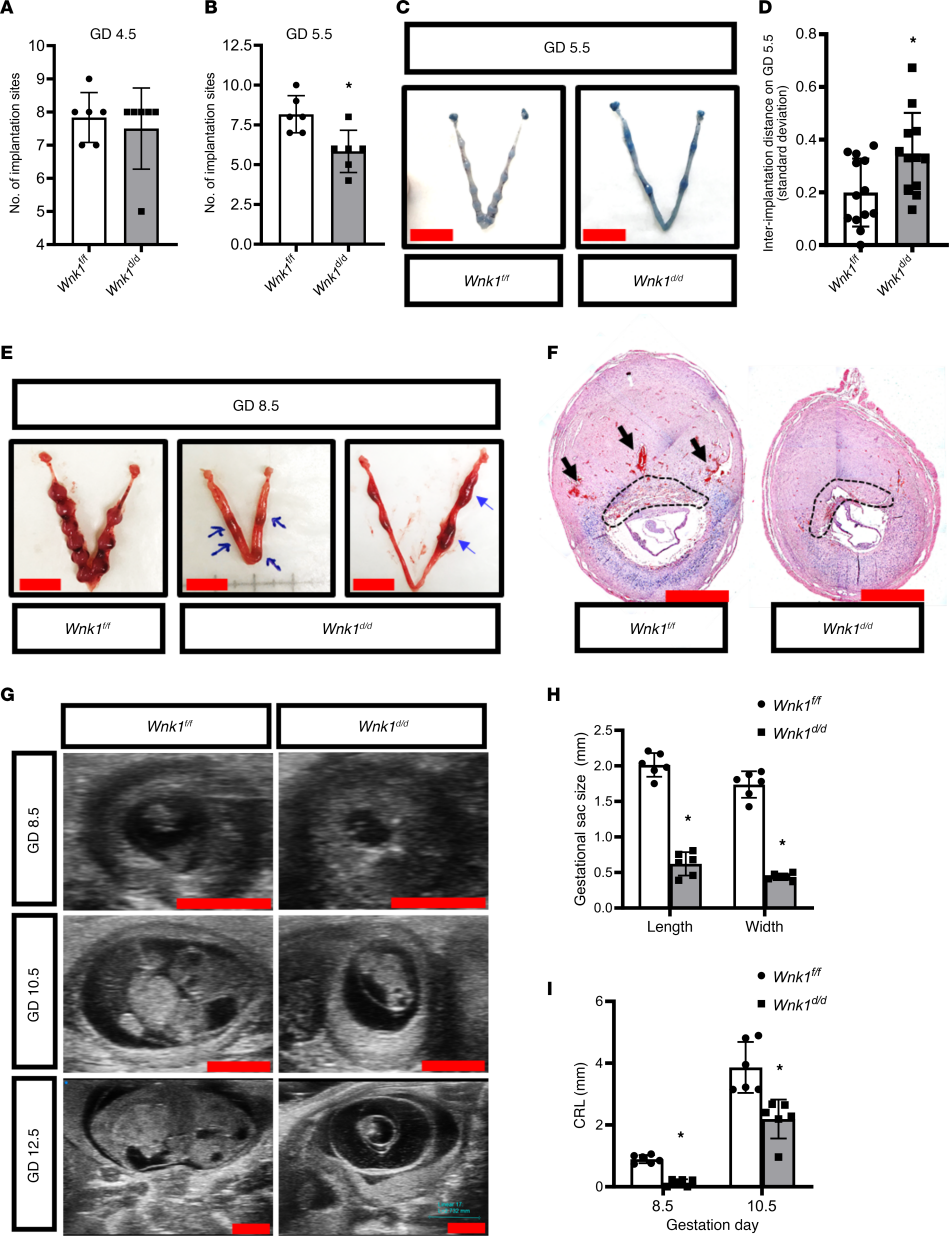
Abnormal embryo development and increased resorption in *Wnk1^d/d^* mice. (**A** and **B**) Number of implantation sites on GD 4.5 (**A**) and GD 5.5 (**B**) in *Wnk1^fl/fl^* and *Wnk1^d/d^* mice (*n* = 6). (**C** and **E**) Uterine gross morphology on GD 5.5 (**C**) and GD 8.5 (**E**), with implantation sites on GD 5.5 marked by Evans blue dye, and blue arrows indicate resorption and abnormal decidualization on GD 8.5. Scale bars: 1 cm. (**D**) Comparison of the standard deviation of interimplantation distance in *Wnk1^fl/fl^* and *Wnk1^d/d^* mice (*n* = ≥12 uterine horns, 7 mice per genotype). (**F**) Hematoxylin and eosin staining of cross section through the center of decidual mass on GD 8.5 from *Wnk1^fl/fl^* and *Wnk1^d/d^* mice, with black arrows and dashed line indicating decidual vessels and placental tissues, respectively. Scale bars: 1 mm. (**G**) Ultrasound scans of uterus and embryo during midpregnancy at GD 8.5, 10.5, and 12.5. Scale bars: 2 mm. (**H** and **I**) Quantification of gestational sac size by length and width on GD 8.5 (**H**), and embryo size by crown-rump length (CRL) on GD 8.5 and 10.5, as measured from ultrasound scans (**I**, *n* = 6). All quantitative results shown are mean ± SD, *P < 0.05. All *t* tests were 2-tailed, Student’s *t* test (**B** and **D**), and Mann-Whitney *U* test (**A**, **H**, and **I**).

Interestingly, for the 29.4% mated *Wnk1^d/d^* mice with embryos implanted at the expected GD 4.5, the number of implantation sites was similar to their *Wnk1^fl/fl^* control littermates (Figure 4A). However, the number of implantation sites present on GD 5.5 was significantly lower in the *Wnk1^d/d^* mice, indicating that the delay in implantation was associated with reduced number of implantation sites (Figure 4, B and C). Additionally, spacing between the implantation sites in the *Wnk1^d/d^* mice was irregular whereas the implantation sites observed in the *Wnk1^fl/fl^* mice were evenly distributed (Figure 4C). This is supported by a significant increase in the standard deviation of interimplantation sites’ distance in *Wnk1^d/d^* uteri compared with *Wnk1^fl/fl^* uteri (Figure 4D). For the *Wnk1^d/d^* mice that were able to implant promptly, implantation spacing was more evenly distributed (Figure 3F), suggesting that the delay may affect both implantation numbers and spacing. Examination of the uterus and embryo during midpregnancy (GD 8.5) further demonstrated that the *Wnk1^d/d^* mice carried either resorbed embryos (Figure 4E, middle) or abnormally formed decidual masses (Figure 4E, right), compared with the normally sized decidual masses observed in the control mice (Figure 4E, left). Moreover, we also observed multiple embryos within 1 decidual zone (Figure 4E, right), possibly from the cluttered/delayed implantation. Morphology was evaluated by examining the cross sections through the center of the decidual mass, which showed that the *Wnk1^fl/fl^* mice have vascularized and initiated placentation (Figure 4F, black arrows and dashed lines, respectively), both of which were lacking in the *Wnk1^d/d^* uteri. Ultrasound scans demonstrated decreased gestation sac size (Figure 4, G and H) and decreased embryo size at both GD 8.5 and GD 10.5 (Figure 4, G and I). By GD 12.5, embryo resorption was frequently observed in the *Wnk1^d/d^* mice (Figure 4G). Collectively, these findings demonstrate that uterine WNK1 ablation led to abnormal implantation and negatively affected embryo development, resulting in the compromised pregnancy outcome and subfertility.

### Loss of uterine WNK1 elevated AKT signaling.

**Figure 5:**
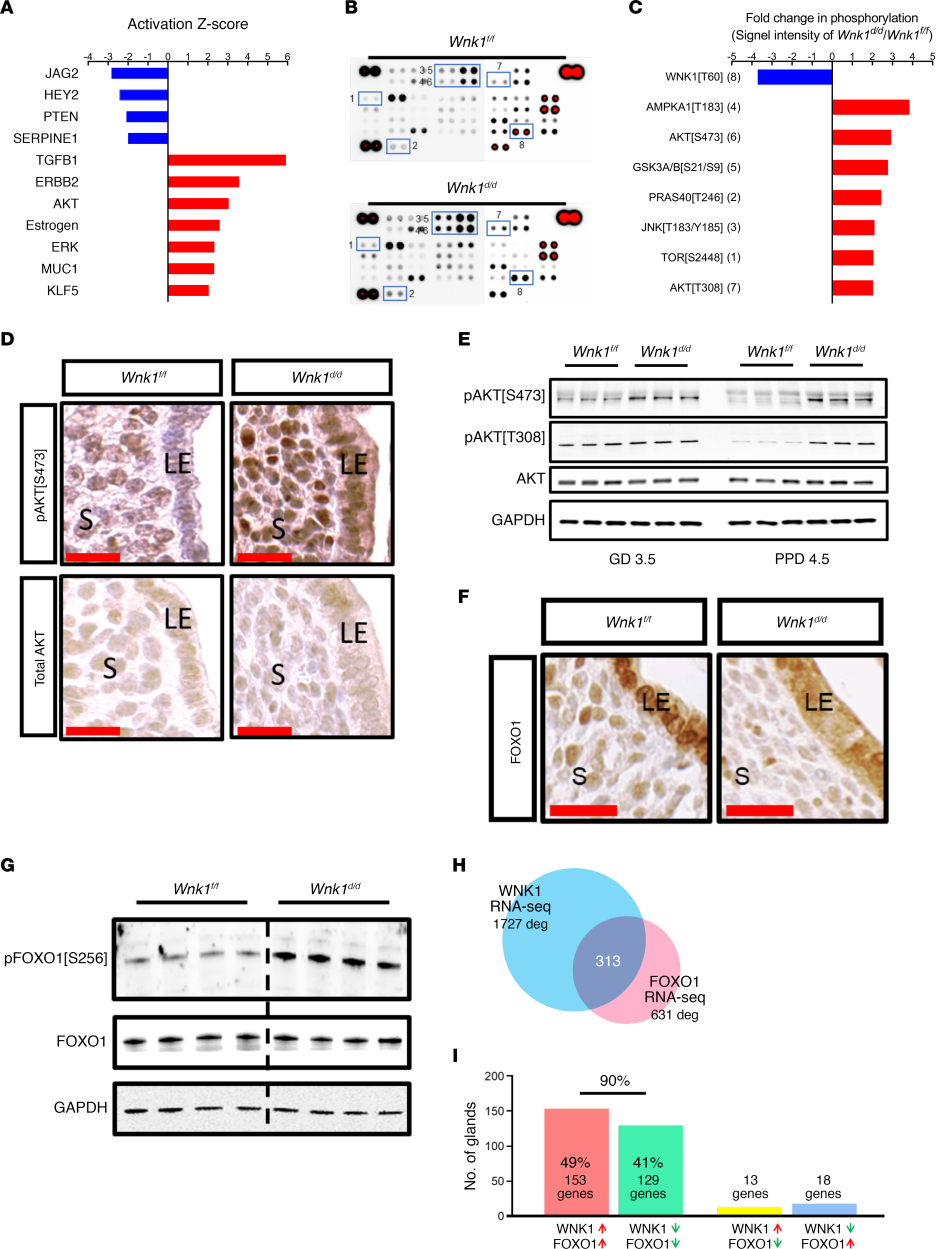
Loss of uterine WNK1-elevated AKT signaling. (**A**) Activity of upstream regulators as predicted by IPA based on the altered uterine transcriptome of *Wnk1^d/d^* mice on PPD 4.5. See Supplemental Table 5 for complete list. (**B** and **C**) Kinome phosphorylation status in *Wnk1^d/d^* and *Wnk1^fl/fl^* uteri on PPD 4.5, with selected alterations shown in **C**. All kinases with more than 1.5 FC in signal intensity as quantified by ImageJ (NIH) are shown in Supplemental Figure 4. Results were acquired using pooled uterine lysate from 6 mice in each group. (**D** and **E**) Expression of phosphorylated and total AKT in *Wnk1^fl/fl^* and *Wnk1^d/d^* uteri on GD 4.5 as shown by IHC, and on GD 3.5 and PPD 4.5 as shown by Western blotting (**E**); scale bars: 25 μm. (**F**) Expression of AKT-regulated implantation marker FOXO1 on GD 4.5 in the stroma and epithelium of *Wnk1^fl/fl^* and *Wnk1^d/d^* mice. Scale bar: 25 μm. (**G**) Western blot analysis showing levels of phosphorylated and total FOXO1 in *Wnk1^fl/fl^* and *Wnk1^d/d^* uteri on PPD 4.5. (**H**) Comparison of DEGs between the uteri of *Wnk1*-ablated mice versus their control littermates (1727 DEGs; blue) and *Foxo1*-ablated mice versus their control littermates (631 DEGs; pink) identified 313 common genes. (**I**) Percentage of the 313 genes categorized into commonly upregulated (pink), commonly downregulated (green), or upregulated in one and downregulated in the other (yellow and blue).

To fully characterize the molecular mechanisms underlying the loss of WNK1-induced implantation defect, we next examined global gene expression profile by RNA-Seq in the uterus during receptivity. To ensure that the analysis was conducted only on the maternal uterine tissues and not the embryos, we used vasectomized WT males to induce pseudopregnancy in the *Wnk1^fl/fl^* and *Wnk1^d/d^* mice. Pregnancy was confirmed by serum progesterone levels on pseudopregnancy day (PPD) 4.5 (Supplemental Table 2). In total, there were 14,423 and 14,337 genes expressed in the *Wnk1^fl/fl^* and *Wnk1^d/d^* uteri, respectively; of which 14,024 were expressed in both. The transcriptomes were subjected to principal components analysis as a measure of quality control, which segregated according to genotype, indicating that the samples were well characterized by genotype (Supplemental Figure 3). Using a defining threshold of *q* value under 0.05 for significance and fold change (FC) over 1.5 as differential expression, we identified 1727 significantly and differentially expressed genes (DEGs) in the *Wnk1^d/d^* uterus during receptivity (Supplemental Table 3). We then conducted detailed analyses to characterize the molecular alterations associated with uterine *Wnk1* ablation using the Database for Annotation, Visualization, and Integrated Discovery (DAVID) bioinformatic database and Ingenuity Pathway Analysis (IPA). Biological processes associated with the DEGs were adhesion, cell movement and locomotion, inflammation, and blood vessel development (Supplemental Table 4). Many important molecular functions associated with implantation were also deregulated in the *Wnk1^d/d^* uteri, such as cell proliferation and apoptosis, Notch signaling, cell differentiation, epithelial to mesenchymal transition, cytokine production, and response to estrogen. Prediction of upstream regulator activity further showed altered activity for many important receptivity mediators, including the suppression of JAG, HEY2, PTEN, and SERPINE1 (Figure 5A). On the other hand, TGFB1, ERBB2, AKT, estrogen, ERK, MUC1, and KLF5 were predicted to show increased activity (Figure 5A, for the complete list see Supplemental Table 5). Because WNK1 is a kinase, we next examined the alterations in the kinase phosphorylation network. Image-based phosphokinase array was employed to evaluate the phosphorylation status of multiple kinases in the uterus during receptivity (Figure 5, B and C). Loss of WNK1 altered the phosphorylation of TOR (MTOR), SRC, PRAS40 (AKT1S1), JNK, AMPKA1 (PRKAA1), GSK3A/B (GSK3A and GSK3B), and AKT (Figure 5, B and C; all kinases with more than 2 FC in phosphorylation are shown in Supplemental Figure 4A). The phosphorylation of AKT, GSK3A/B, and PRAS40 were independently validated via Western blotting (Supplemental Figure 4B), and all showed elevated phosphorylation in *Wnk1^d/d^* uteri during receptivity. Interestingly, AKT has been identified as an activated upstream regulator by IPA, which coincided with its increased phosphorylation. This was confirmed in both the epithelium and the stroma via IHC (Figure 5D). Indeed, we found that in the control mice, phosphorylation of AKT was suppressed as the mice transitioned into the receptive phase from GD 3.5 to PPD 4.5; however, the *Wnk1^d/d^* mice maintained high AKT phosphorylation both before and during receptivity (Figure 5E).

The AKT-regulated transcription factor FOXO1 is an indispensable mediator of implantation because mice lacking uterine FOXO1 expression experience infertility due to failed implantation (25). Interestingly, AKT is known to phosphorylate FOXO1, resulting in its nuclear exclusion and thereby inhibiting its transcriptional activity (26, 27). FOXO1 expression was assayed using IHC, and we found that, indeed, there was a marked decrease in its nuclear form in both the luminal epithelium and underlying stroma (Figure 5F). This was further confirmed by the increase in the levels of phosphorylated FOXO1 in the *Wnk1^d/d^* uteri (Figure 5G). Because FOXO1 nuclear exclusion prevents its transcriptional activity, we compared WNK1-regulated genes and FOXO1-regulated genes in the uterus during the receptive phase (25). This revealed that roughly half of the FOXO1-regulated genes were also deregulated in *Wnk1^d/d^* uteri (Figure 5H), and strikingly, 90% of those genes were deregulated in the same direction under WNK1- and FOXO1-deficient conditions — these included known implantation- and decidualization-associated genes, such as *Msx2*, *Wnt5a*, and *Muc1* (Figure 5I, Supplemental Table 3, and Vasquez et al.; refs. 25, 28–30). These findings indicate that uterine loss of WNK1 led to elevated AKT phosphorylation and, in turn, increased FOXO1 phosphorylation and nuclear exclusion, hence altering expression of FOXO1-regulated genes.

**Figure 6:**
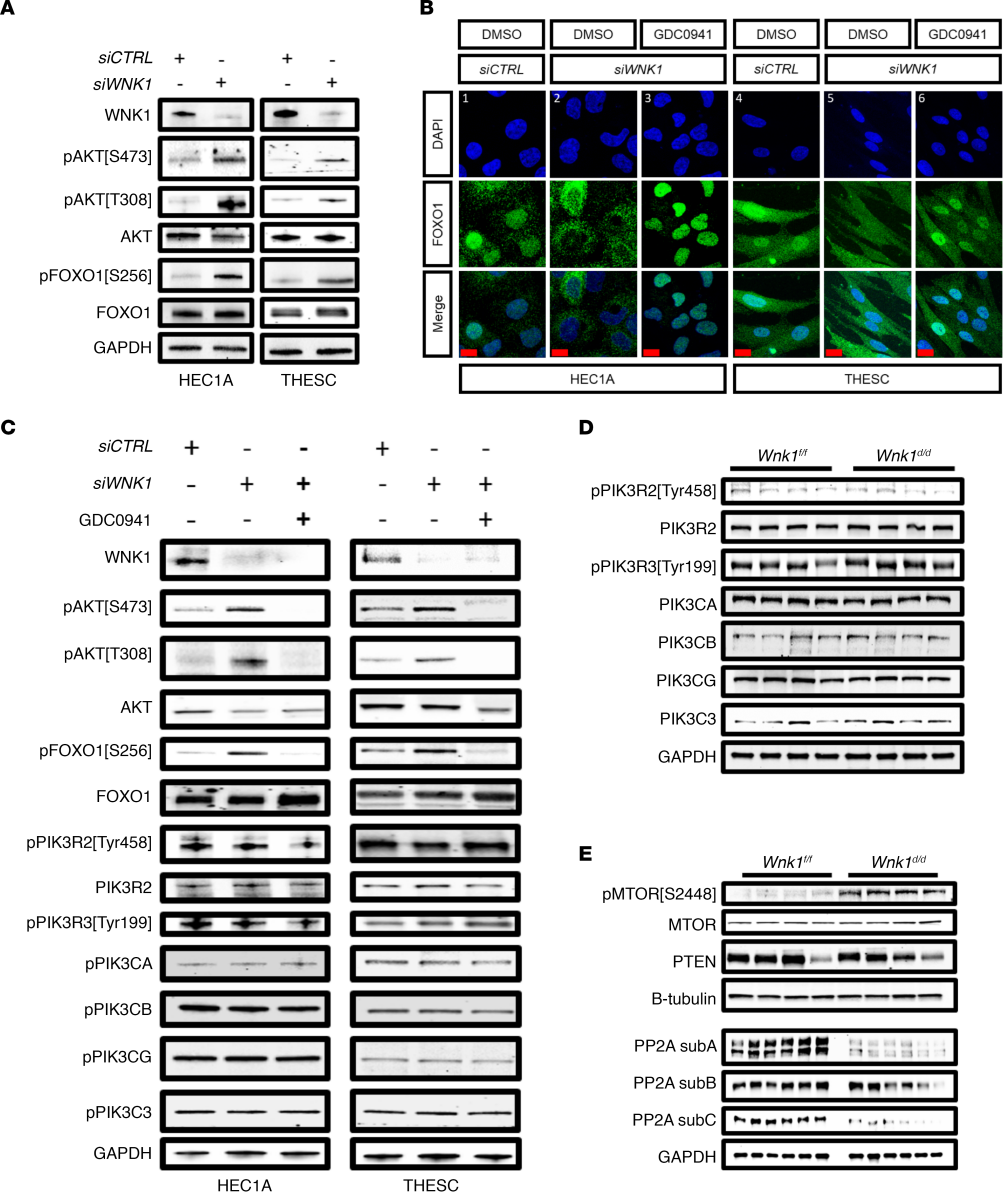
*WNK1* ablation led to FOXO1 nuclear exclusion via AKT phosphorylation, which was associated with decreased PP2A phosphatase expression. (**A**) Western blot showing levels of phosphorylated and total AKT and FOXO1 in HEC1A cells and THESCs transfected with 48 nM small interfering control (*siCTRL*) or *siWNK1*. (**B**) Immunofluorescence showing FOXO1 subcellular localization (green), with nuclei presented in DAPI in HEC1A and THESC control cells (columns 1, 4), *siWNK1*-transfected cells (columns 2, 5), and GDC0941-treated, *siWNK1*-transfected cells (columns 3, 6); scale bars: 20 μm. (**C**) Expression of FOXO1, AKT, and PI3K members in HEC1A cells and THESCs transfected with *siCTRL* or *siWNK1* and treated with AKT inhibitor GDC0941. (**D** and **E**) Expression of PI3K proteins (**D**) and MTOR, PP2A subunits, and PTEN (**E**) in *Wnk1^fl/fl^* and *Wnk1^d/d^* uteri on PPD 4.5.

*WNK1 regulates FOXO1 localizationviaAKT, which is associated with decreased protein phosphatase 2A expression and activity*. We next examined whether the WNK1-AKT-FOXO1 regulatory axis was maintained in human endometrial HEC1A cells (epithelial) and transformed human endometrial stromal cells (THESCs) (stromal). WNK1 protein attenuation using small interfering RNA against *WNK1* (*siWNK1*) induced AKT and FOXO1 phosphorylation in both cell lines (Figure 6A). In order to test whether AKT facilitated FOXO1 localization downstream of WNK1, we next treated these cells with an AKT inhibitor, GDC0941, and examined whether it could rescue WNK1 ablation–induced phosphorylation and nuclear exclusion of FOXO1. FOXO1 localization clearly decreased in the nucleus of both cells after transfection with *siWNK1* (Figure 6B, columns 1 vs. 2 and 4 vs. 5). However, when the *siWNK1*-transfected cells were treated with GDC0941, nuclear FOXO1 was readily restored (Figure 6B, columns 3 and 6). This suggested that WNK1 inhibition–induced nuclear exclusion of FOXO1 is mediated through AKT. This is further supported by the findings that AKT inhibition rescued WNK1 knockdown–induced FOXO1 phosphorylation (Figure 6C). Interestingly, GDC0941 treatment reduced the phosphorylation of AKT and FOXO1 to a level that is lower than that seen in the *siCTRL*-transfected, untreated cells (considered basal level). Because GDC0941 inhibits AKT through its upstream regulator PI3K (31), it is likely that PI3K lies upstream of WNK1 in regulating AKT. Indeed, none of the PI3K family members were affected by WNK1 inhibition, including PIK3CA, PIK3CB, and PIK3CG, as well as phosphorylation levels of PIK3R2 and PIK3R3 (Figure 6C). Similarly, no changes in the expression of these proteins was observed between the *Wnk1^fl/fl^* and *Wnk1^d/d^* mice during receptivity (Figure 6D).

Potential mechanisms by which WNK1 regulates AKT activity were explored by searching the upstream regulators predicted by IPA. Several candidates, including PTEN, PPP2CA, and sirolimus (rapamycin, Supplemental Table 5), were identified. PTEN and PPP2CA are both phosphatases that regulate AKT phosphorylation, and both displayed repressed activities in the *Wnk1^d/d^* mice during receptivity (*z* scores of –2.079 and –1.195, respectively, Supplemental Table 5). Sirolimus is an MTOR inhibitor, which was strongly inhibited (*z* score of –2.95, Supplemental Table 5). We found that MTOR phosphorylation and protein phosphatase 2A (PP2A) subunits A and C were altered in the *Wnk1^d/d^* mice, while PTEN level was not significantly different (Figure 6E). This finding suggested that increased AKT phosphorylation in the *Wnk1^d/d^* mice may be mediated through elevated MTOR or repressed PP2A activity. Because MTOR is both a regulator and a substrate of AKT (32, 33), we examined whether WNK1 ablation–induced AKT phosphorylation is mediated through MTOR. We inhibited MTOR activity using rapamycin and examined AKT/FOXO1 phosphorylation as well as FOXO1 localization as a readout of AKT activity. As shown in Supplemental Figure 5A, rapamycin treatment did not reverse the nuclear exclusion of FOXO1 induced by WNK1 inhibition. Additionally, AKT and FOXO1 phosphorylation was not rescued by rapamycin treatment (Supplemental Figure 5B). Similar results were observed in HEC1A cells, where WNK1 and MTOR double knockdown failed to rescue AKT and FOXO1 phosphorylation (Supplemental Figure 5C). Thus, MTOR is likely not the WNK1 mediator controlling AKT activity, and its elevated phosphorylation is a result of elevated AKT activity, rather than its cause.

### WNK1 regulates AKT phosphorylation through direct interaction with PPP2R1A.

We next explored the possible regulatory link between WNK1 and PP2A/AKT using an unbiased WNK1 immunoprecipitation–mass spectrometry (IP-MS) approach to identify WNK1 binding partners. Successful WNK1 IP was confirmed by examining the lysate for WNK1 expression after IP using a rabbit IgG (negative control) or WNK1 targeting antibody from HEC1A cells (Supplemental Figure 6), and the peptides identified by MS are listed in Supplemental Table 6. Among those were peptides belonging to WNK1 itself, as well as a known WNK1 substrate, OXSR1/OSR1 (12), confirming the validity of the pull-down results (Supplemental Table 6).

**Figure 7:**
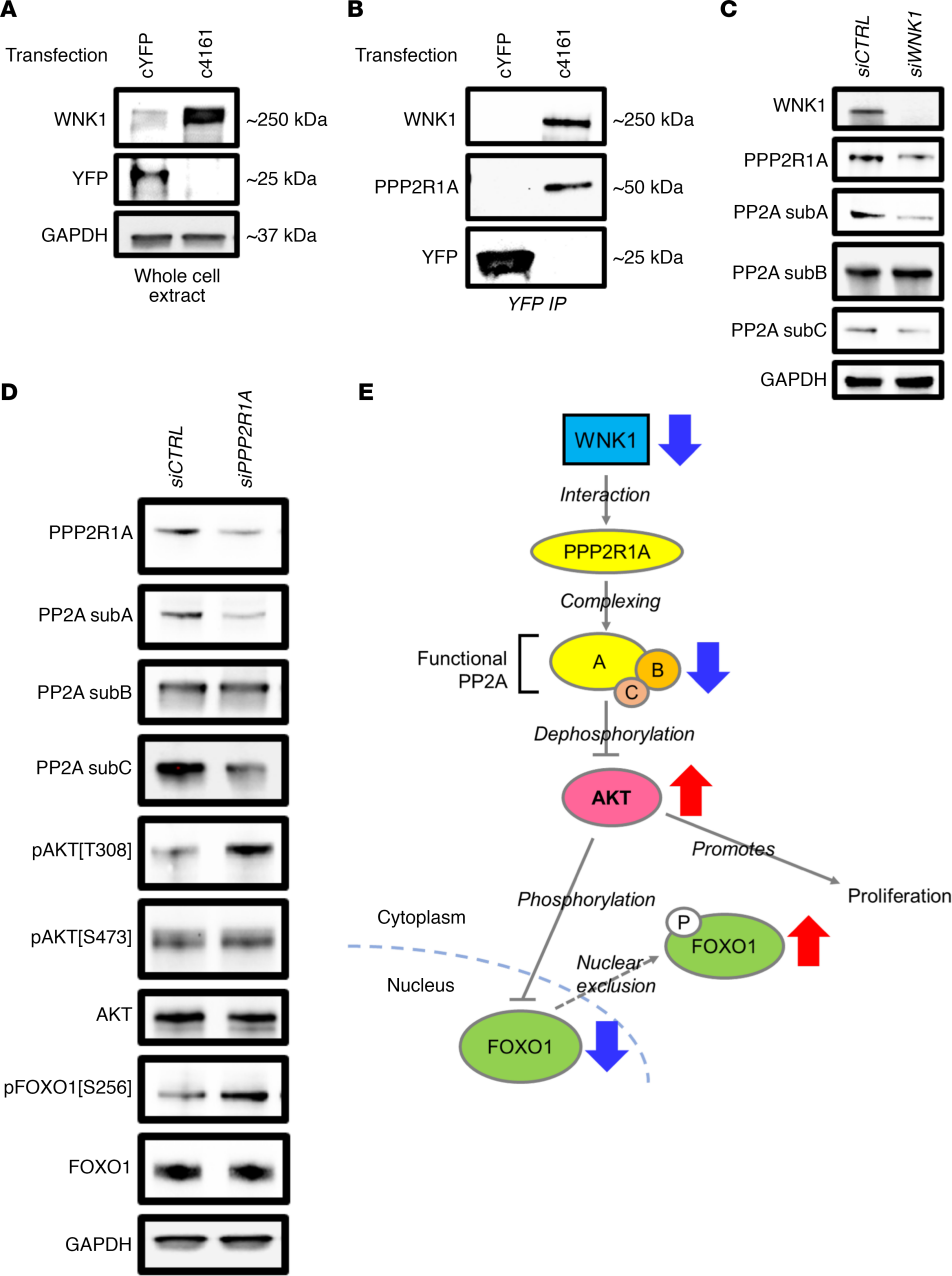
WNK1 regulated AKT signaling through direct interaction with PPP2R1A. (**A**) WNK1 and YFP expression in HEC1A cells transfected with the YFP-expressing control plasmid (cYFP) or YFP-tagged WNK1 expression construct (c4161). (**B**) Coimmunoprecipitation of WNK1 and PPP2R1A with YFP from HEC1A whole cell lysate, as indicated by Western blotting. (**C**) Expression of PPP2R1A and PP2A subunits in HEC1A cells transfected with 24 nM *siCTRL* or *siWNK1* for 72 hours. (**D**) Expression of PP2A subunits A, B, and C; AKT; and FOXO1 in HEC1A cells transfected with 72 nM *siCTRL* or siPPP2R1A for 72 hours. (**E**) Diagram illustrating the WNK1/PP2A/AKT/FOXO1 signaling axis. WNK1 physically interacts with PPP2R1A, the alpha isoform of the scaffold subunit that forms the functional PP2A subunit. PP2A negatively regulates AKT, and AKT negatively regulates FOXO1 by phosphorylation and nuclear exclusion. AKT also promotes epithelial cell proliferation. As indicated by the blue and red arrows, decreased or loss of WNK1 will then lead to decreased PP2A activity, AKT hypersignaling, and increased cytoplasmic FOXO1 retention and epithelial proliferation.

Putative WNK1 binding proteins included Wnt regulators (OFD1 and CCDC88C), chromosome modulating and DNA repair proteins (SMCA1, KIF11, FANCI, RAD50, and SLC25A5), and proteins associated with the endoplasmic reticulum and ribosomal functions (UGGT1, SEC23A, HYOU1, EMC1, AIFM1, HM13, SCFD1) as well as the mitochondria (AIFM1, SLC25A5). Of particular interest were the components of protein phosphatase complexes PP2A (PPP2R1A), and PP6 (PPP6R3), as both are known AKT regulators (34, 35). Because the enzymatic activity of PP2A was predicted by IPA as repressed in the *Wnk1^d/d^* mice (PPP2CA, Supplemental Table 5), we postulated that interaction between WNK1 and PPP2R1A (the alpha isoform of the scaffold subunit A of PP2A) affects PP2A activity. In order to confirm the interaction of WNK1 and PPP2R1A, a yellow fluorescent protein–tagged (YFP-tagged) WNK1 (c4161, Supplemental Figure 7) was expressed in HEC1A cells, then immunoprecipitated using a YFP nanobody, followed by detection for PPP2R1A in the pull-down. We first confirmed that c4161 transfection induced exogenous WNK1 expression when compared with the control cells transfected with YFP-only expressing construct (cYFP, Figure 7A). WNK1 was subsequently detected in the lysate immunoprecipitated for YFP (Figure 7B, upper panel), which coimmunoprecipitated with PPP2R1A (Figure 7B, middle panel).

We next explored the biological implications of this WNK1-PPP2R1A interaction. The PP2A phosphatase complex is composed of the scaffold subunit A with 2 isoforms, the regulatory subunit B with 13 isoforms and the enzymatic subunit C with 2 isoforms. As shown earlier, uterine WNK1 ablation led to decreased protein levels of subunits A and C (Figure 6E), yet RNA-Seq showed no alteration in transcription of the 4 genes encoding these 2 subunits (*Ppp2ca*, *Ppp2cb*, *Ppp2r1a*, and *Ppp2r1b*) in the *Wnk1^d/d^* mice. It has been reported that the stability of the PP2A subunits depends on their association with each other (36). Hence, reduced subunit levels could be an indication that the complexing capacity of the subunits was reduced in the absence of WNK1, leading to their degradation. We therefore postulated that the WNK1-PPP2R1A interaction is necessary for the association of the PP2A subunits. To test this idea, we examined the levels of PPP2R1A, total PP2A subunits A and C in WNK1-knockdown HEC1A cells, and accordingly found their reduced levels upon WNK1 inhibition (Figure 7C). Last, to validate that PP2A mediates AKT/FOXO1 signaling, we inhibited *PPP2R1A* expression in HEC1A cells using siRNA and examined the components of the PP2A-AKT-FOXO1 signaling axis. As expected, PPP2R1A knockdown caused a reduction in total subunits A and C of PP2A (Figure 7D). Interestingly, AKT phosphorylation was selectively induced on threonine 308, but not serine 473, after PPP2R1A knockdown (Figure 7D). This, nonetheless, translated to elevated FOXO1 phosphorylation, indicating that loss of PP2A activity–induced AKT phosphorylation on this residue alone was sufficient to increase FOXO1 phosphorylation (Figure 7D). These findings confirmed that in endometrial cells, WNK1 controls AKT activity through modulating its phosphorylation, which is partially mediated through PP2A (Figure 7E). As such, loss of WNK1 led to decreased PP2A activity and increased AKT phosphorylation, resulting in pathological outcomes associated with AKT hypersignaling, such as hyperplasia and FOXO1 deregulation (Figure 7E, blue and red arrows).

## Discussion

Reproductive biology has relied profoundly on transcriptomic analyses to identify novel players that may serve crucial functions in the regulation of fertility. While this approach has uncovered many key components in the reproductive tract, it is unable to detect alterations at the proteomic level, such as posttranslational modifications (PTMs). In many cases, the PTMs control protein activity and stability and hence are the actual determinants of functional output. Through a proteomic approach, we identified WNK1 as a potential regulator of uterine biology with previously unreported reproductive functions (2).

In this study, we explored this further by examining the in vivo function of WNK1, and we demonstrate here that loss of WNK1 led to hyperplasia, adenomyosis-like features, and impaired implantation. We show for the first time to our knowledge that WNK1 robustly repressed AKT activity and that loss of WNK1 led to increased AKT phosphorylation and signaling. This was evident through the increased MTOR and FOXO1 phosphorylation (26, 27, 33), resulting in nuclear exclusion of the latter and disrupted embryo implantation (25). Although both uterine WNK1- and FOXO1-deleted mice shared phenotypic similarity of implantation impairment, the 2 mouse models did not completely phenocopy each other. Ablation of WNK1 resulted in chronic epithelial hyperplasia and adenomyotic glands, which were absent with FOXO1 ablation. Furthermore, while uterine loss of WNK1 and FOXO1 shared a common transcriptomic footprint, each had a unique set of altered genes. Specifically, WNK1 regulated an additional 1414 genes accounting for the epithelial hyperplasia and myometrial invasion of the glands, which were not observed in the FOXO1 mice (25).

AKT is well known for promoting cell proliferation and is a target in anticancer therapy (37), which is supported by our observation that *Wnk1^d/d^* mice displayed epithelial hyperplasia resulting from escalated proliferation. Evidence has also shown a link between adenomyosis- and estrogen-induced AKT overactivity (38). Although not the focus of this work, the cellular changes and molecular events associated with WNK1 deficiency–induced adenomyotic gland invasions seem to parallel observations made in humans, including excessive ECM deposition, elevated *Moesin* expression, and AKT hypersignaling (19, 21). In the future, *Wnk1^d/d^* mice could serve as an ideal model system to study adenomyosis, which affects a significant proportion of the population (39). Interestingly, there is evidence in the literature showing that WNK1 is a substrate of AKT in other cellular and animal systems (40, 41). We observed a reduction in WNK1 phosphorylation after AKT inhibition in HEC1A cells and THESCs (data not shown), suggesting that AKT could reciprocally interfere with WNK1 activity. These, together with our demonstration that WNK1 inhibits AKT phosphorylation and activity in endometrial cells, suggest that the WNK1-AKT relationship involves a negative feedback and is likely more complex than previously thought.

Given that 30% of the *Wnk1^d/d^* mice were able to implant promptly with normal numbers of embryos, we rarely observed normal sized litters from these mice. This suggested that there must exist other impairments in subsequent pregnancy development accounting for the compromised fertility. The significant proportion of *Wnk1^d/d^* mice succumbing to pregnancy complications, including death near term and dystocia, which indicated poor support of pregnancy and impaired uterine muscle contractility. This could be attributed to impaired decidualization; indeed, our previous in vitro study demonstrated that WNK1 is a regulator of decidualization (3). We did not extensively characterize decidualization in this study due to the preceding implantation defect, which complicates decidualization data interpretation. However, transcriptomic analysis identified alterations in several decidualizing regulators, including Notch (HEY2 and JAG2), ERK, and MUC signaling (42–44). Therefore, we are confident in speculating that loss of WNK1 likely negatively affected decidualization in vivo. Additionally, the premature loss of fertility in those mice that survived to the end of the breeding trial suggested that the postpartum tissue repair and remodeling may also be affected by loss of WNK1. Interestingly, Zhu et al. reported on the AKT-dependent endometrial stromal cell repair in humans (45), providing a possible explanation for the premature sterility.

We identified a regulatory link between WNK1 and AKT with PP2A as the intermediate. Here, the loss of WNK1 reduced PP2A subunits A and C as well as PP2A phosphatase activity in endometrial cells of both humans and mice. A possibility is that WNK1 facilitates the binding of the subunits, resulting in PP2A complex binding and stabilization (36). Mechanistically, WNK1 directly interacts with PPP2R1A and hence this interaction may be crucial for PP2A complex formation; however, further experimentation will be necessary to test this hypothesis. It is worth noting that there must be additional mechanisms through which WNK1 is repressing AKT, as *PPP2R1A* knockdown in human cells restored only the phosphorylation on T308. However, WNK1 ablation induced phosphorylation on both T308 and S473.

As above mentioned, uterine ablation of WNK1 exhibited pleiotropic effects including epithelial hyperplasia and adenomyotic features. Although neither is cancerous, both are progressive conditions, which may lead to malignant transformation (46, 47). Functional interpretation of the transcriptome reiterated this, where many signaling pathways associated with cancer development and progression were altered in the *Wnk1^d/d^* uteri, including elevated TGFB, AKT, and estrogen (47–50). Strikingly, a recurrent mutation of *PPP2R1A* is associated with serous endometrial carcinoma (51, 52), and this mutation has been found to affect oncogenic signaling through a dominant negative effect (53). Although we demonstrate here that WNK1 positively regulated PP2A activity and propose a possibility that WNK1 could be associated with endometrial cancer, its exact role in endometrial cancer is yet unknown and worth exploring. Interestingly, there exists a known *WNK1* mutation in humans that causes *WNK1* overexpression, and no impact on reproductive health has been reported. Nonetheless, investigating the activity of WNK1 in human endometrial cancers would shed light on its potential role in this disease. In summary, we demonstrate that WNK1 is critical in maintaining normal uterine morphology and in mediating epithelial homeostasis and implantation and may play a potential role in human endometrial pathogenesis.

## Methods

### Generation of transgenic mice.

The *Wnk1^fl/fl^* mice with the insertion of *loxP* sites into the 5′ and 3′ region of exon 2 were provided in-house (University of Iowa Healthcare, Iowa City, Iowa, USA) (14). The *Wnk1^fl/fl^* mice were crossed to mice carrying *Cre* under the control of the progesterone receptor (*PGR^Cre^*) to generate conditional uterine *Wnk1*-ablated mice (*Wnk1^d/d^*, Supplemental Figure 1) (16).

### Fertility trial.

Seven- to 11-week-old *Wnk1^fl/fl^* and *Wnk1^d/d^* mice were housed with WT C57BL/6J or CD1 males (The Jackson Laboratory) for a period of 6 months. The mice were monitored daily for pregnancy and delivery. Upon the first observation of delivery, the total number of pups was counted.

### Implantation determination and pseudopregnancy.

Virgin mice of age 6–10 weeks were housed with WT C57BL/6J males and monitored each morning until vaginal plug was observed. The first noon following the observation of the vaginal plug was defined as GD 0.5. Mice were anesthetized by isoflurane inhalation on GD 4.5 and 5.5, followed by retro-orbital administration of 200 μL of 1% Evans blue dye to visualize the implantation status after euthanization. For mice sacrificed on GD 4.5 and showing no visible implantation site, uterine horns were flushed with PBS, and the eluant was examined under a bright-field microscope to determine presence of blastocysts. Uterine horns were fixed for 48 hours in 4% paraformaldehyde (PFA) for histology and IHC or flash-frozen for RNA and protein extraction. For RNA-Seq, pseudopregnancy was induced by mating the females to vasectomized WT male mice, and all procedures were conducted as described above.

### Superovulation assay.

Three-week-old virgin *Wnk1^fl/fl^* and *Wnk1^d/d^* mice were subjected to a superovulation regimen, which consisted of a pregnant mare’s serum gonadotropin (5 IU, i.p.) (493-10-2.5, Lee Biosolutions), followed by human chorionic gonadotropin (5 IU, i.p.) (869031, MilliporeSigma) 48 hours later. Mice were placed with WT CD1 males overnight. Mating was confirmed by presence of vaginal plug the next morning (GD 0.5), and mice were euthanized on GD 1.5 for oviduct flushing. The number of embryos was determined by counting under a bright-field microscope.

### Serum collection.

On GD or PPD 4.5, mice were anesthetized by intraperitoneal administration of Fetal Plus (1 mg/10 g body mass, Vortech Pharmaceuticals, Ltd), and whole blood was collected via retro-orbital puncture. Serum was collected by allowing the blood to clot at room temperature (RT) for 30 minutes, then centrifuged at 1000*g* for 10 minutes at 4°C. Hormone assays included estradiol (ES180S-100, Calbiochem ELISA) and progesterone (IB79105, IBL ELISA). These were conducted by the Ligand Core Laboratory of University of Virginia, Center for Research in Reproduction.

### High-frequency ultrasound imaging.

On GDs 8.5, 10.5, and 12.5, high-frequency ultrasound imaging was used to evaluate the uterus and embryo development. Dams were anesthetized by isoflurane inhalation and placed onto an electric heating pad to maintain body temperature. Abdominal hair was removed using depilatory cream (Nair, Church & Dwight Co., Inc.), and eye lubricant was applied to prevent desiccation. Dams were manipulated into a supine position for the scan while heart rate and body temperature were continuously monitored. Images were visualized and captured using the VevoR 2100 Imaging System with a 550s scan head (FUJIFILM VisualSonics Inc.) at 55 MHz.

### Tissue processing, histology, and immunohistochemical and immunofluorescence staining.

After PFA fixation, tissues were placed in 70% ethanol for a minimum of 48 hours, followed by dehydration, paraffin embedding, and sectioning to 5 μm thickness. Sections were deparaffinized by 3 serial incubations in Citrisolv clearing agent (22-143-975, Thermo Fisher Scientific) and rehydrated through decreasing ethanol dilutions. Histological sections were subjected to hematoxylin and eosin and Masson’s trichrome staining, followed by dehydration through increasing ethanol dilutions, with incubation in Citrisolv before mounting. For IHC, sections were subjected to antigen retrieval after rehydration using the VECTOR Laboratories Antigen Unmasking Solution as per manufacturer’s instructions (H-3300). Blocking of endogenous peroxidase was performed by treating the sections with 3% H_2_O_2_ for 10 minutes at RT. Tissues were blocked in 5% normal donkey serum (NDS) for 60 minutes, then incubated with primary antibody at 4°C overnight. The slides were washed twice in PBS, and secondary antibody diluted in 1% *w/v* bovine serum albumin (BSA) was applied. The ABC reagent was applied to tissue according to the manufacturer’s instructions (PK-6100, VECTOR Laboratories). Signals were developed using the VECTOR Laboratories DAB ImmPACT Staining Kit (SK-4105). Finally, the tissue sections were counterstained with hematoxylin and dehydrated through increasing ethanol concentration, followed by Citrisolv incubation and mounting. For immunofluorescence, tissue sections were subjected to antigen retrieval as described above, then blocked in 0.4% *v/v* Triton X-100, 1% BSA, and 5% NDS for 30 minutes followed by overnight incubation in primary antibody prepared in 0.4% Triton X-100/PBS at 4°C. Sections were washed in PBS and incubated with secondary antibodies diluted in 0.4% Triton X-100/PBS for 90 minutes. Finally, slides were washed 3 times in PBS and coverslipped in DAPI-containing mounting medium (H-1400, Vector Laboratories). Details of antibodies used in this study are provided in Supplemental Table 7.

### RNA extraction and cDNA conversion.

Frozen tissues were disrupted in TRIzol (Invitrogen, Thermo Fisher Scientific) by bead milling, followed by 2 aqueous phase separations using 1-Bromo-3-chloropane and chloroform (MilliporeSigma). Pure ethanol was added to the aqueous layer, and the RNA was extracted using the QIAGEN RNeasy RNA Mini Prep Kit columns as per manufacturer’s instructions (74104, QIAGEN). Resulting RNA concentration and quality were determined using NanoDrop ND-1000. cDNA was generated by reverse transcription using the M-MLV Reverse Transcriptase (28025013, Thermo Fisher Scientific) following the manufacturer’s instructions.

### qRT-PCR.

qRT-PCR was performed using the SsoAdvanced Universal SYBR Green Supermix (1725274, Bio-Rad) with the following primers (from 5′ to 3′; F, forward, and R, reverse): *Wnk1* AGGCAGAGATTCAAAGAAGAGG (F) and CCCAGGAATCATAGAATCGAACA (R); *Msn* CCATGCCGAAGACGATCA (F) and CCAAACTTCCCTCAAACCAATAG (R); and *Foxa2* GAGACTTTGGGAGAGCTTTGAG (F) and GATCACTGTGGCCCATCTATTT (R). *Lif* expression was determined using the TaqMan Master Mix (Life Technologies, Thermo Fisher Scientific) and TaqMan probes (Applied Biosystems, Thermo Fisher Scientific). The ΔΔCt values were calculated using 18S RNA control amplification results to acquire the relative mRNA expression for each gene.

### RNA-Seq.

For each mouse, 1 μg of uterine RNA was sent to the NIH Intramural Sequencing Center to create a library using the TruSeq RNA Kit (Illumina) following the manufacturer’s instructions. The libraries were sequenced with a HiSeq 2000 System (Illumina). The raw RNA reads (75 nt, paired end) were processed by filtering with average quality score greater than 20. Reads that passed the initial processing were aligned to the mouse reference genome (mm10; Genome Reference Consortium Mouse Build 38 from December 2011) using TopHat version 2.0.4 (54). Expression values of RNA-Seq were expressed as fragments per kilobase of exon per million fragments (FPKM). Differential expression was calculated using Cuffdiff function from Cufflinks version 2.2 (55). Transcripts with the average FPKM > 1 in at least 1 group, *q* < 0.05, and at least 1.5-fold difference in FPKM were defined as DEGs. Data for this publication have been deposited in NCBI’s Gene Expression Omnibus and are accessible through GEO Series accession number GSE144802. Functional annotation for the DEGs derived from RNA-Seq were analyzed by IPA and DAVID (56).

### Human phosphokinase antibody array.

Site-specific phosphorylation levels of 43 kinases were measured using the Human Phospho-Kinase Array (HPA) kit (ARY003B, R&D Systems, Bio-Techne) according to the manufacturer’s instructions with the experimental design as described below. Mice were euthanized on PPD 4.5, with uterine tissues flash frozen and stored at –80°C. Lysates were extracted independently from 6 mice per group by bead milling in the HPA lysis buffer, and protein concentrations were determined using the BCA Kit (23225, Pierce, Thermo Fisher Scientific). Equal amounts from each mouse were pooled in each group (to a total of 900 μg), and the remaining steps followed the standard HPA protocol. Signal intensity was quantified by ImageJ (NIH) (57). Images shown in the main figure were chosen to allow visualization of maximal difference between *Wnk1^fl/fl^* and *Wnk1^d/d^* mice for selected kinases, but quantification was performed using blots in the nonsaturation range.

### Protein extraction from uterine tissues and protein expression analysis.

Tissues were homogenized in RIPA Lysis and Extraction Buffer (89900, Thermo Fisher Scientific) supplemented with protease inhibitor cocktail (11836170001, Roche Diagnostics) and phosphatase inhibitor cocktail (4906837001, Roche Diagnostics), then centrifuged at 10,000*g* for 10 minutes at 4°C and pellets discarded. Protein concentrations were measured using the BCA Kit (23225, Pierce, Thermo Fisher Scientific). Heat-denatured protein samples were resolved using 7.5%, 10%, or gradient 4%–20% Criterion Tris-HCl precast gels (Bio-Rad), followed by transferring using the Trans-Blot Turbo Transfer System (Bio-Rad), as per the manufacturer’s instructions. PVDF and nitrocellulose membranes were used for target proteins more than 200 kDa and less than 200 kDa, respectively. After transfer, the membranes were blocked in 5% *w/v* nonfat milk or BSA. Membranes were incubated with primary antibody at 4°C overnight, washed 3 times, and incubated in secondary antibody the next day. Finally, membranes were washed another 3 times, and depending on the expected signal strength, different peroxidase chemiluminescent substrates were used: KPL LumiGLO^R^ (546101, Seracare), Clarity Western ECL Substrate (1705060, Bio-Rad), and Amersham ECL Prime Western Blotting Detection Reagent (RPN2232, GE Healthcare Life Sciences). Antibody sources and dilutions are summarized in Supplemental Table 7. For each Western blot, GAPDH or B-tubulin were detected as the loading control, and in cases where the target protein was in the same region as the loading control proteins, a duplicate gel was run and transferred in parallel. For each set of samples, a representing GAPDH or B-tubulin blot is shown.

### Tissue clearing and 3D reconstruction.

Uterine tissues were fixed in 4% PFA for 16 hours, followed by 3 rinses in PBS. Tissues were incubated in hydrogel monomer solution AP40 (4% *v/v* acrylamide and 0.25% *w/v* VA-044 in PBS) for 72 hours at 4°C protected from light. Oxygen was then removed in a chamber connected to vacuum and nitrogen, followed by incubation at 37°C for 3 hours to initiate tissue-hydrogel hybridization. Hydrogel was removed from the tissues via 3 PBS washes, and tissues were subsequently incubated in 8% SDS prepared in PBS for 7 days at 37°C with shaking, and the SDS solution was replaced twice during incubation. Tissues were washed 5 times for 1 hour in PBS and blocked in 5% NDS prepared in PBS/Triton X-100 with 0.01% of sodium azide. The samples were incubated in primary antibody in 2% *v/v* NDS and 0.01% *w/v* sodium azide for 6 days at RT with constant rotation, followed by five 1-hour washes in 0.1% *v/v* Triton X-100 in PBS (PBS-T). Secondary antibody was similarly prepared and incubated for another 6 days at RT with constant rotation and protected from light, with antibody replaced after 3 days. Finally, the samples were washed an additional 5 times for 1 hour in PBS-T and incubated in Refractive Index Matching Solution (80% *w/v* Histodenz, D2158, MilliporeSigma; prepared in 0.02 M phosphate buffer, pH 7.5, with 0.1% Tween-20 and 0.01% sodium azide, refractive index = 1.46) for 1–3 days, and samples were mounted in fresh Reflective Index Mounting Solution using a 1 mm deep iSpacer (SUNJin Lab). Details of antibodies used in this study are provided in Supplemental Table 7.

### Cell culture.

Human endometrial epithelial cell line HEC1A and telomerase-THESCs were obtained from American Type Culture Collection (Rockville, Maryland, USA). HEC1A cells were cultured in McCoy’s 5A modified medium (16600082, Gibco, Thermo Fisher Scientific), and the THESCs were maintained in DMEM/F12 (1:1) (11330-032, Gibco, Thermo Fisher Scientific), both supplemented with 10% FBS (10437-028, Gibco, Thermo Fisher Scientific) and 100 U/mL penicillin and 100 μg/mL streptomycin, unless otherwise stated.

### siRNA transfection and drug treatments.

Cells were transfected with siRNAs using the Lipofectamine RNAiMax transfection reagent (13778150, Thermo Fisher Scientific) following the manufacturer’s protocol. Cells were transfected with 24–72 nM siRNA in transfection medium supplemented with 2% charcoal-stripped FBS (12676-029, Gibco, Thermo Fisher Scientific) for 24-48 hours before replacing with fresh growth medium. Proteins were harvested from cells 72 hours after transfection unless otherwise stated. The siRNAs used in this study were nontargeting siRNA (*siCTRL*, D-001810-10-20, Dharmacon), *WNK1* targeting siRNA (*siWNK1*, L-005362-02-0005, Dharmacon), *MTOR* targeting siRNA (*siMTOR*, L-003008-00-0005, Dharmacon), and *PPP2R1A* targeting siRNA (*siPPP2R1A*, L-060647-00-0005, Dharmacon). AKT and MTOR inhibitors GDC0941 and rapamycin (S1065 and S1039, respectively, Selleckchem) were dissolved in DMSO, and cells were treated with 5 μM GDC0941 and 10–40 μM rapamycin for 24 hours, while the control cells received equivalent volumes of DMSO.

### Immunofluorescence of cultured cells.

Cells were seeded in 4-chambered cover glass (155382, Thermo Fisher Scientific) and followed with siRNA transfection and/or drug treatment. Cells were rinsed in PBS, fixed in 4% PFA, and permeabilized in 0.5% Triton X-100 and PBS for 10 and 5 minutes, respectively. Cells were incubated in blocking buffer (5% *v/v* NDS; 0.2% *v/v* fish gelatin, G7765, MilliporeSigma; 0.2% *v/v* Tween-20 in PBS) for 30 minutes at 37°C. Primary antibody was diluted in blocking buffer and added to the cells for 60 minutes, followed by secondary antibody for another 60 minutes; both incubation steps were performed at 37°C in a humidified chamber. Finally, cells were rinsed 3 times with 0.2% Tween-20/PBS and coverslipped using a DAPI-containing mounting medium (H-1400, VECTOR Laboratories). Details of antibodies used in this study are provided in Supplemental Table 7.

### WNK1 IP-MS.

HEC1A cells were grown to 70% confluence, followed by collection using trypsin. Cells were washed 2 times in cold PBS, followed by resuspension in cell lysis buffer (50 mM Tris-HCl pH 7.5, 150 mM NaCl, 1 mM EDTA, 1% NP-40, 1% sodium deoxycholate, 0.1% SDS, with protease and phosphatase inhibitors added fresh to 1×). Cells were incubated on ice for 10 minutes, followed by sonication on medium power (3 × 5 seconds). Lysate was centrifuged at 13,000*g* for 10 minutes at 4°C. WNK1 targeting antibody was added at 1:100 to the supernatant and incubated with rotation at 4°C overnight. Prewashed beads (50% protein A and 50% protein G, 10002D and 10004D, respectively, Thermo Fisher Scientific) were added to the immunocomplex and incubated for 30 minutes at RT with rotation. Beads were pelleted using a magnetic separation rack, followed by 3 washes in lysis buffer. Beads were heated to 100°C with SDS buffer (150 mM Tris-HCl pH 6.8, 6% SDS, 0.3% bromophenol blue, 30% glycerol, 3% B-mercaptoethanol) for 5 minutes, before electrophoresis through a 7.5% Criterion Tris-HCl precast gel (Bio-Rad). Gel regions containing the proteins were excised and minced, and digests were performed with a ProGest robotic digester (Genomic Solutions) where the gel pieces were destained by incubation in 25 mM ammonium bicarbonate with 50% acetonitrile (*v/v*) twice for a total of 30 minutes. The gel pieces were dehydrated in acetonitrile, followed by drying under a nitrogen stream, and further incubated with 250 ng trypsin (Promega) for 8 hours at 37°C. The digests were collected, and peptides were re-extracted 3 times. The extractions were pooled for each sample, lyophilized and resuspended in 20 μL 0.1% formic acid. The protein digests were analyzed by liquid chromatography/MS (LC/MS) on a Q Exactive Plus mass spectrometer (Thermo Fisher Scientific) interfaced with a nanoAcquity ultraperformance liquid chromatography system (Waters Corporation), equipped with a 75 μm × 150 mm BEH dC18 column (1.8 μm particle, Waters Corporation) and a C18 trapping column (18 μm × 20 mm) with a 5 μm particle size at a flow rate of 400 nL/min. The trapping column was positioned in line with the analytical column and upstream of a micro-tee union, which was used both as a vent for trapping and as a liquid junction. Trapping was performed using the initial solvent composition. A volume of 5 μL of digested sample was injected into the column, and peptides were eluted by using a linear gradient from 99% solvent A (0.1% formic acid in water *v/v*) and 1% solvent B (0.1% formic acid in acetonitrile *v/v*), to 40% solvent B over 60 minutes. For the mass spectrometry, a data-dependent acquisition method was employed with an exclusion time of 15 seconds and an exclusion of +1 charge states. The mass spectrometer was equipped with a NanoFlex source and was used in the positive ion mode. Instrument parameters were as follows: sheath gas, 0; auxiliary gas, 0; sweep gas, 0; spray voltage, 2.7 kV; capillary temperature, 275°C; S-lens, 60; scan range (*m/z*) of 200 to 2000; 2 *m/z* isolation window; resolution: 70,000; automated gain control, 2 × 10^5^ ions; and a maximum injection time of 200 ms. Mass calibration was performed before data acquisition using the Pierce LTQ Velos Positive Ion Calibration mixture (Thermo Fisher Scientific). Peak lists were generated from the LC/MS data using Mascot Distiller (Matrix Science), and the resulting peak lists were searched using the Spectrum Mill software package (Agilent) against the SwissProt database. Searches were performed using trypsin specificity and allowed for 1 missed cleavage and variable methionine oxidation. Mass tolerances were 20 ppm for MS scans and 50 ppm for MS/MS scans.

### Generation of mammalian YFP-WNK1 expression constructs.

The coding region of the WNK1 sequence (NM_014823.3) with attL sites and N-terminal TEV cleavage site was synthesized by GeneWiz Inc. and cloned into pUC57 (Kanamycin) plasmid. Gateway Cloning with LR Clonase II mix (Thermo Fisher Scientific) was used to transfer the WNK1 sequence into the Vivid Colors pcDNA6.2/N-YFP vectors (Thermo Fisher Scientific), which created the mammalian expression vectors with YFP fused to the N-terminal end of WNK1 (Supplemental Figure 7, c4161).

### Coimmunoprecipitation.

HEC1A cells were transfected with cYFP or c4161 for 48 hours, followed by trypsinization, 3 washes, and resuspension in lysis buffer (50 mM Tris pH 8.0, 400 mM NaCl, 0.1% NP-40, and 0.5 mM DTT, with protease and phosphatase inhibitors freshly added to 1×). The lysate was incubated at 4°C with rotation for 30 minutes, then centrifuged at 21,100*g* for 10 minutes. The supernatant was added to 1.5 volumes of 25% glycerol, followed by centrifugation at 21,100*g* for 10 minutes at 4°C. Anti-GFP resin slurry was added to the supernatant and nutated for 1 hour at 4°C. Beads were centrifuged at 1000*g* for 5 minutes, 4°C, followed by 6 washes in 100 μL of PBS-T in Bio-Spin columns (7326204, Bio-Rad). The bound immunocomplexes were eluted via 0.1 M glycine, pH 2.0, and eluent was neutralized using 2 M Tris-HCl, pH 8.0.

### Confocal microscopy.

All fluorescence images presented in this study were captured using the ZEISS LSM 780 UV confocal microscope.

### Statistics.

GraphPad Prism versions 7 and 8 were used for data analysis. Each set of data points was first subjected to normality test. Two-tailed Student’s *t* tests and Mann-Whitney *U* tests were performed for normally distributed data and non-normally distributed data, respectively. For percentage of mice with implantation after mating, Fisher’s exact test was performed. In each case, a *P* value less than 0.05 was considered significant.

### Study approval.

All procedures were approved by the following ethics committee: the University of North Carolina at Chapel Hill under IRB file 05-1757. Written, informed consents were obtained from all patients before participation.

All animal studies were conducted in accordance with the *Guide for the Care and Use of Laboratory Animals*, as published by the NIH (National Academies Press, 2011). Animal protocols were approved by the Animal Care and Use Committee of the National Institute of Environmental Health Sciences (NIEHS, protocol numbers 2015-0012 and 2015-0023). The mice were housed with a maximum of 5 per cage with a 12-hour light/12-hour dark cycle and fed irradiated Teklad global soy protein–free extruded rodent diet (Harlan Laboratories, Inc.) and fresh water ad libitum. Euthanization was carried out by carbon dioxide inhalation followed by cervical dislocation. Because there appeared to be premature loss of fertility in the *Wnk1^d/d^* mice, all experiments were conducted following first mating of virgin mice, unless otherwise stated.

## Author contributions

RPAC, SPW, and FJD conceived the study; RPAC and FJD designed the methodology; RPAC validated the results; RPAC and TW performed formal data analysis; RPAC investigated; SLY, JPL, CLH, and FJD provided resources; RPAC and TW curated data; RPAC wrote the original draft; RPAC and FJD reviewed and edited the draft; RPAC visualized the data; FJD supervised the project; RPAC and FJD performed project administration; and FJD acquired funding.

## Supplementary Material

supplemental data

supplemental Table 1

supplemental Table 2

supplemental Table 3

supplemental Table 4

supplemental Table 5

supplemental Table 6

supplemental Table 7

## Version 1 10/13/2020

In-Press Preview

## Version 2 11/19/2020

Electronic publication
