# Supplementary material for: WNK1 regulates uterine homeostasis and its ability to support pregnancy
Source: JCI Insight. 2020 Nov 19;5(22):e141832. doi: 10.1172/jci.insight.141832 (PMC7710275; doi:10.1172/jci.insight.141832)
Supplement: supplemental data [file jciinsight-5-141832-s130.pdf]

WNK1 Regulates Uterine Homeostasis and its Ability to Support Pregnancy

141832-JCI-RG-1

Chi et al., 2020

Supplemental Figures

Figure S1

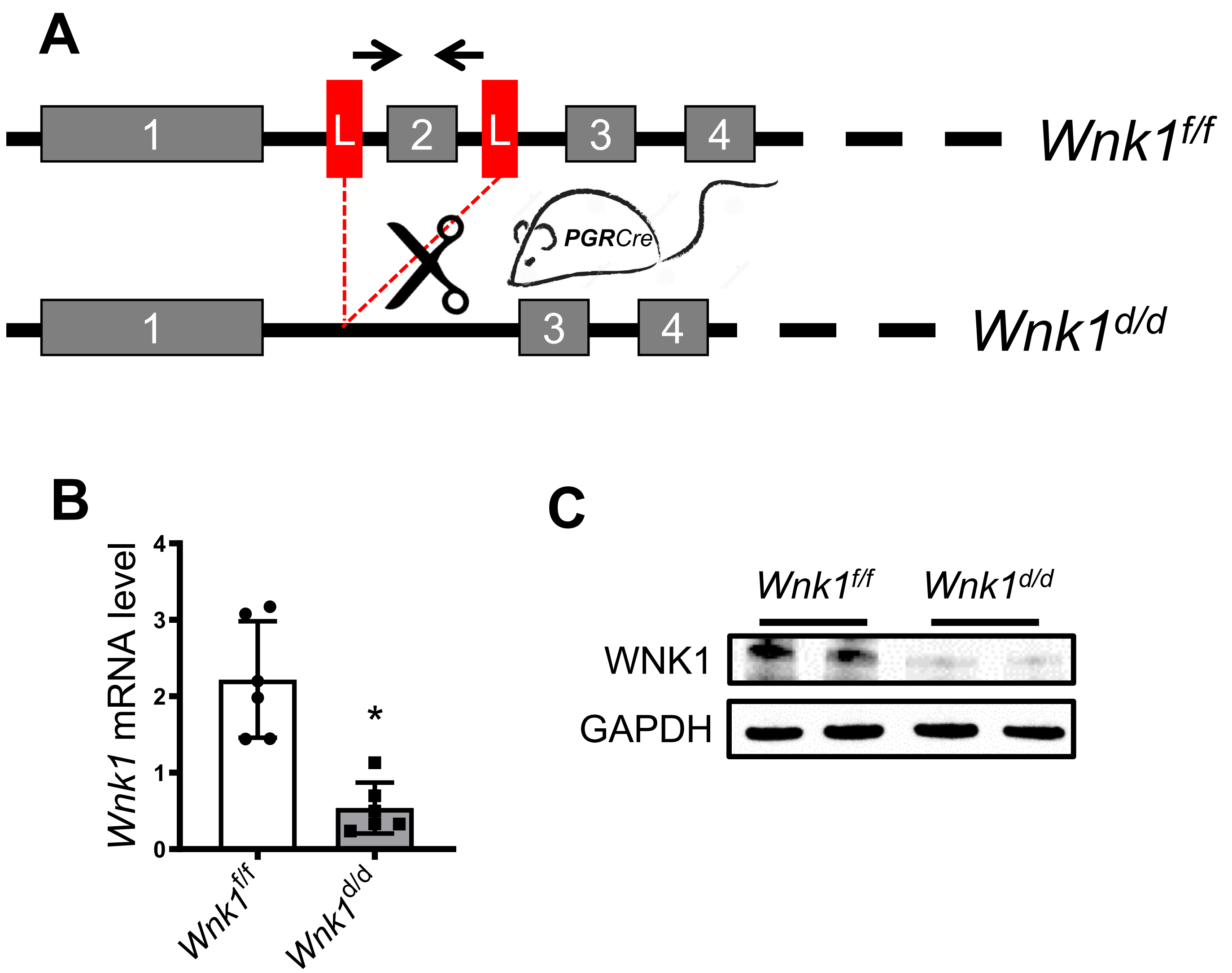
A
1
L
2
L
3
4
Wnk1f/f
PGRCre
1
3
4
Wnk1d/d
B
Wnk1 mRNA level
4
3
2
1
0
*
Wnk1f/f
Wnk1d/d
C
Wnk1f/f
Wnk1d/d
WNK1
GAPDH

Figure S2

A

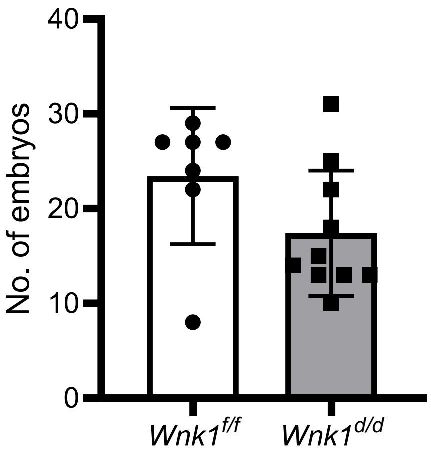

B

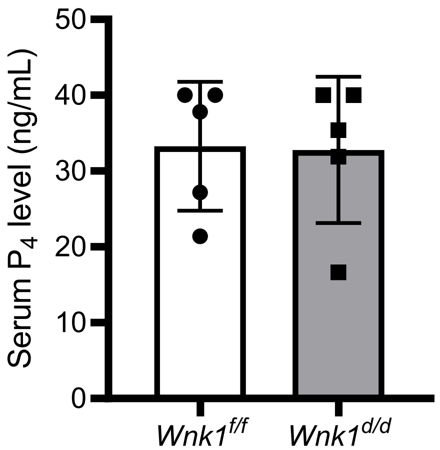

C

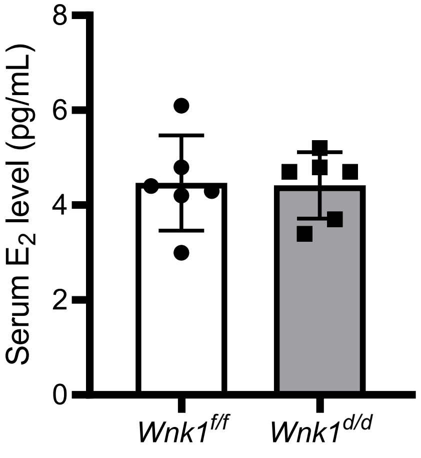

Figure S3

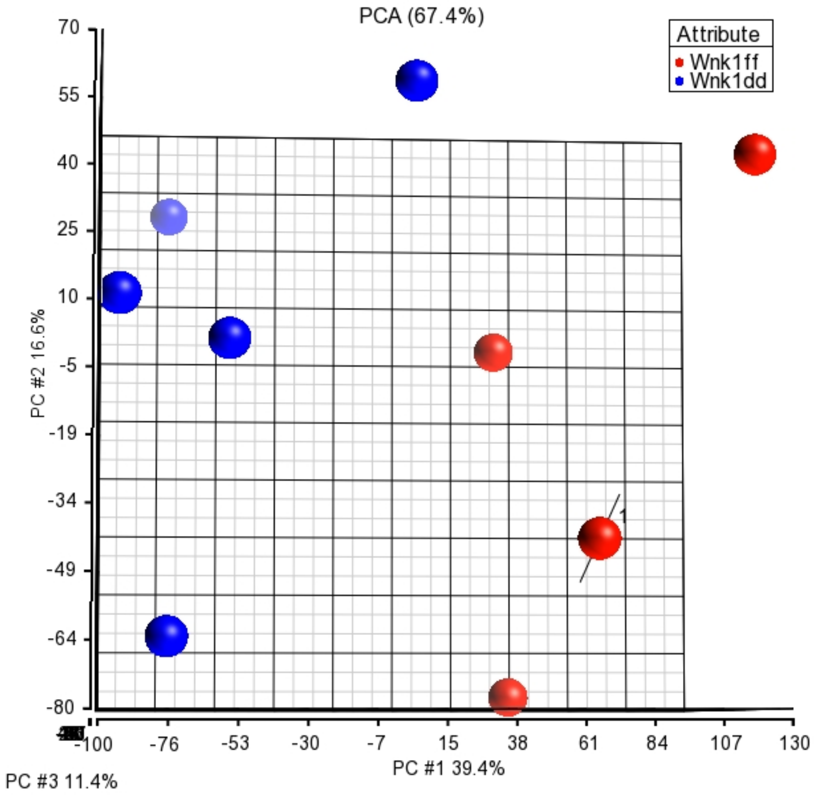

PCA (67.4%)
Attribute
Wnk1ff
Wnk1dd
70
55
40
25
10
-5
-19
-34
-49
-64
-80
PC #2 16.6%
-100
-76
-53
-30
-7
15
38
61
84
107
130
PC #1 39.4%
PC #3 11.4%

Figure S4

A

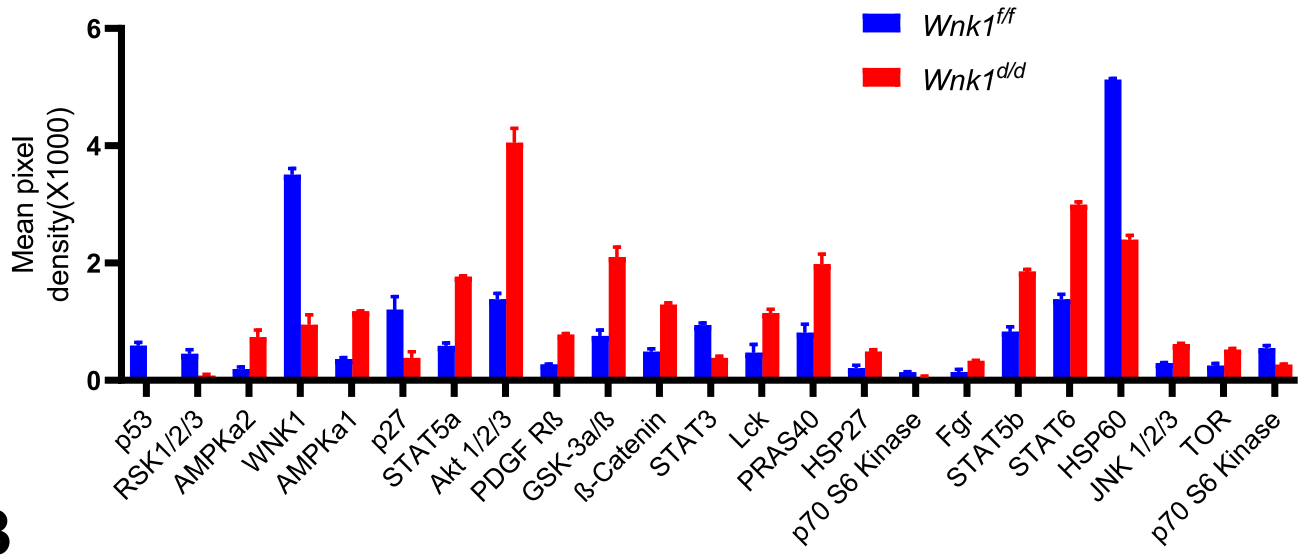

Wnk1f/f
Wnk1d/d
Mean pixel density(X1000)
6
4
2
0
p53
RSK1/2/3
AMPKa2
WNK1
AMPKa1
p27
STAT5a
Akt 1/2/3
PDGF Rß
GSK-3a/ß
ß-Catenin
STAT3
Lck
PRAS40
HSP27
p70 S6 Kinase
Fgr
STAT5b
STAT6
HSP60
JNK 1/2/3
TOR
p70 S6 Kinase

B

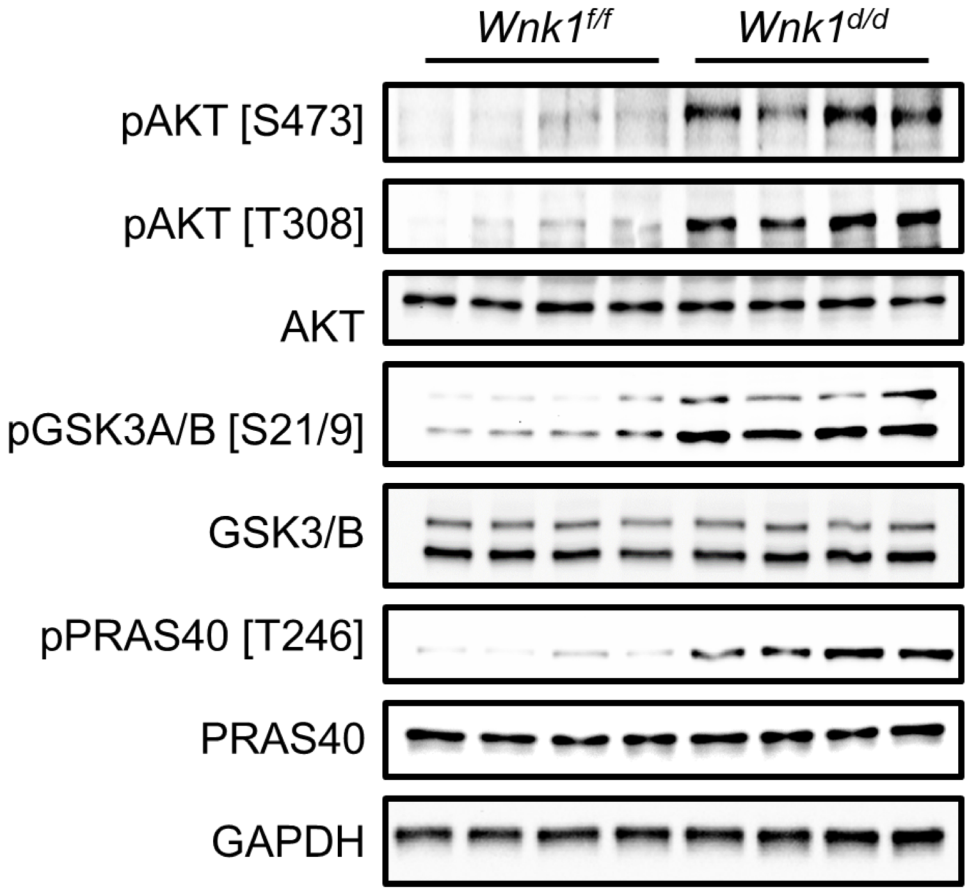

Wnk1f/f
Wnk1d/d
pAKT [S473]
pAKT [T308]
AKT
pGSK3A/B [S21/9]
GSK3/B
pPRAS40 [T246]
PRAS40
GAPDH

Figure S5

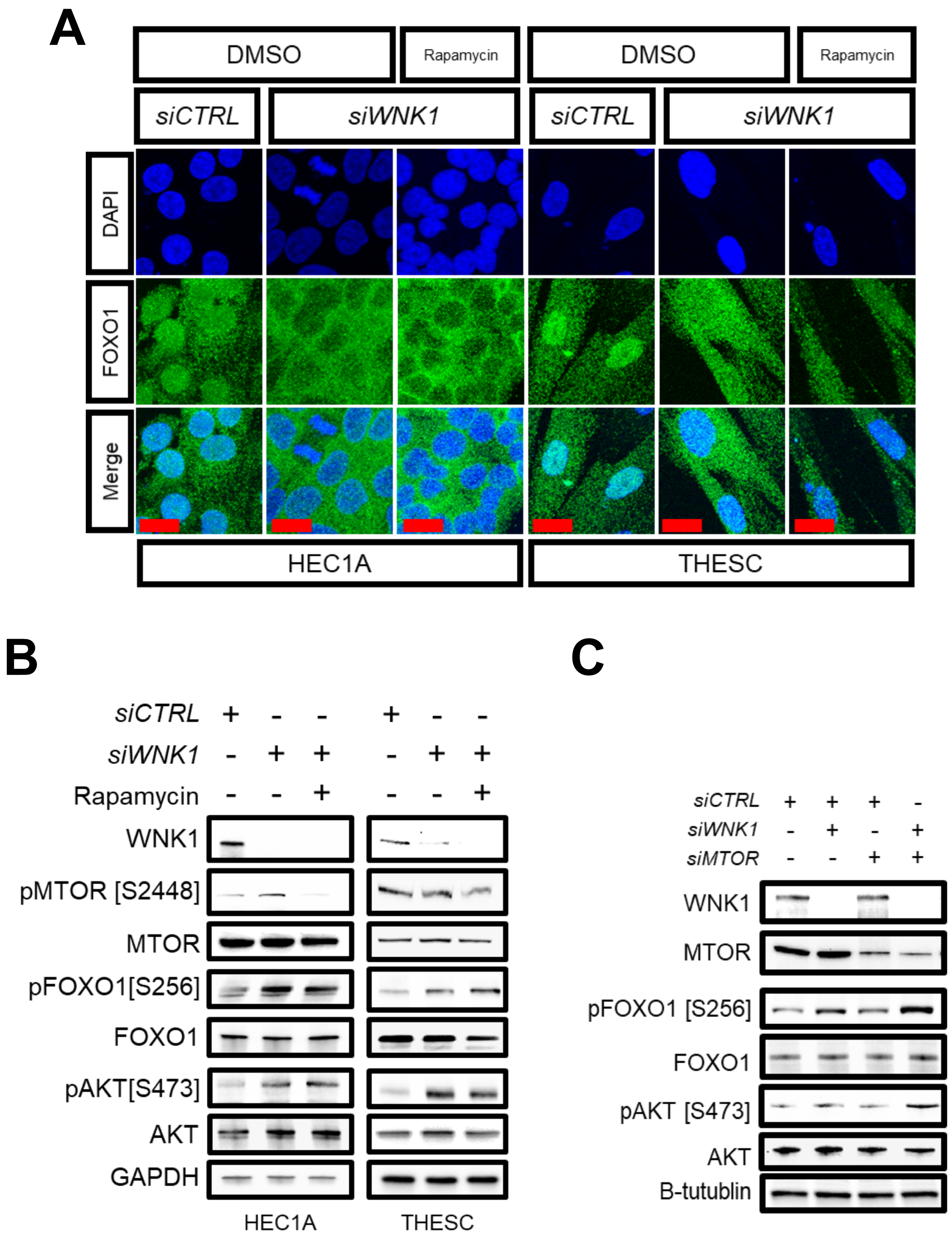
A
DMSO
Rapamycin
DMSO
Rapamycin
siCTRL
siWNK1
siCTRL
siWNK1
DAPI
FOXO1
Merge
HEC1A
THESC
B
siCTRL + - - + - -
siWNK1 - + + - + +
Rapamycin - - + - - +
WNK1
pMTOR [S2448]
MTOR
pFOXO1[S256]
FOXO1
pAKT[S473]
AKT
GAPDH
HEC1A
THESC
C
siCTRL + + + -
siWNK1 - + - +
siMTOR - - + +
WNK1
MTOR
pFOXO1 [S256]
FOXO1
pAKT [S473]
AKT
B-tutublin

Figure S6

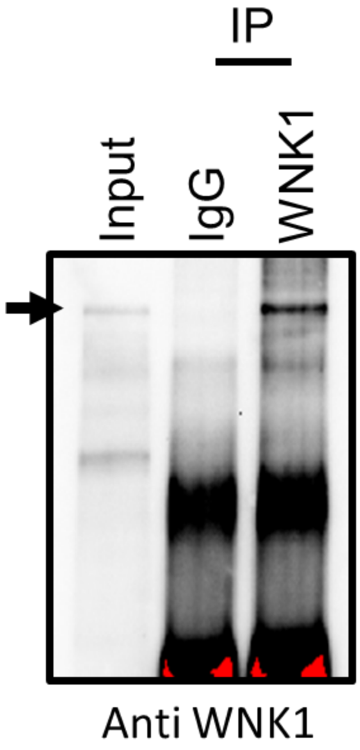

Figure S7

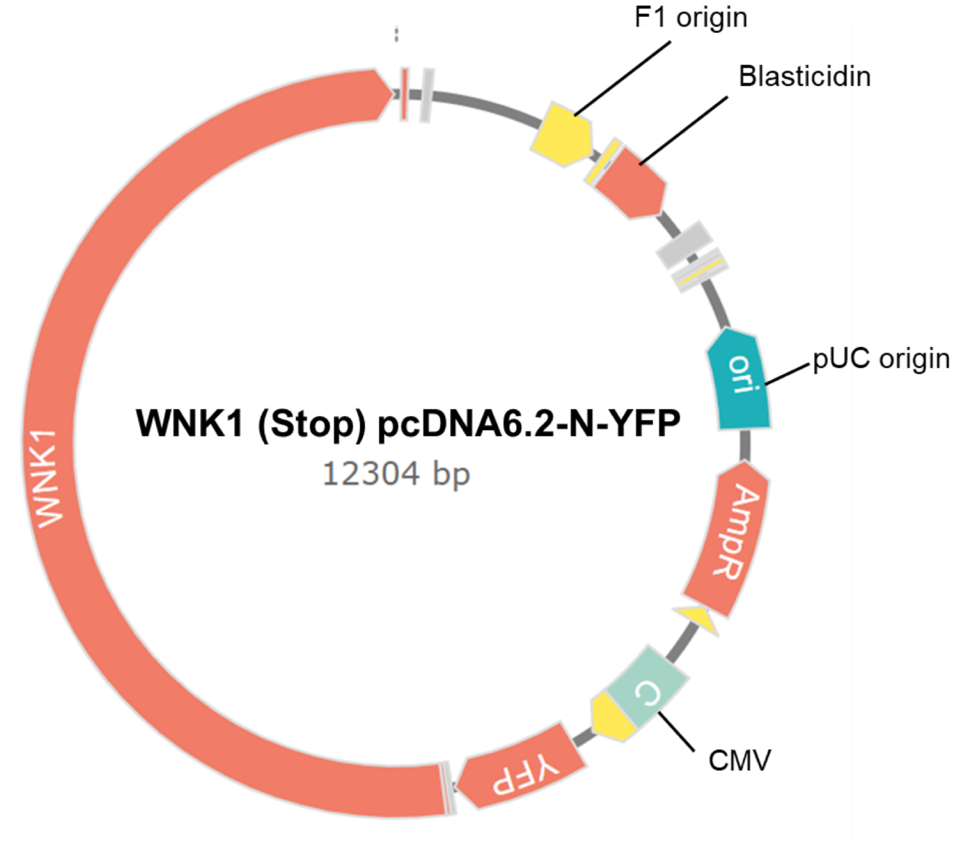
F1 origin
Blasticidin
pUC origin
ori
WNK1 (Stop) pcDNA6.2-N-YFP
12304 bp
WNK1
AmpR
C
CMV
YFP
